# Genome-Wide Study of YABBY Genes in Upland Cotton and Their Expression Patterns under Different Stresses

**DOI:** 10.3389/fgene.2018.00033

**Published:** 2018-02-07

**Authors:** Zhaoen Yang, Qian Gong, Lingling Wang, Yuying Jin, Jianping Xi, Zhi Li, Wenqiang Qin, Zuoren Yang, Lili Lu, Quanjia Chen, Fuguang Li

**Affiliations:** ^1^Xinjiang Research Base, State Key Laboratory of Cotton Biology, Xinjiang Agricultural University, Urumqi, China; ^2^Institute of Cotton Research, Chinese Academy of Agricultural Sciences, Anyang, China

**Keywords:** polarity, *Gossypium hirsutum*, abiotic stress, ovule, transcription factor, segmental duplication

## Abstract

Members of the YABBY gene family, a small plant-specific family of genes, have been proposed to function in specifying abaxial cell fate. Although to date little has been learned about cotton YABBY genes, completion of the cotton genome enables a comprehensive genome-wide analysis of YABBY genes in cotton. Here, a total of 12, 12, and 23 YABBY genes were identified in *Gossypium arboreum* (2n = 26, A_2_), *G. raimondii* (2n = 26, D_5_), and *G. hirsutum* (2n = 4x = 52, [AD]_t_), respectively. Sequence analysis showed that the N-terminal zinc-finger and C-terminal YABBY domains in YABBY proteins are highly conserved among cotton, *Arabidopsis*, and rice. Eighty-five genes from eight sequenced species naturally clustered into five groups, and the *YAB2*-like group could be divided into three sub-groups, indicating that YABBYs are highly conserved among the examined species. Orthologs from the At and Dt sub-genomes (where “t” indicates tetraploid) showed good collinearity, indicating that YABBY loci are highly conserved between these two sub-genomes. Whole-genome duplication was the primary cause of upland cotton YABBY gene expansion, segmental duplication played important roles in YABBY gene expansion within the At and Dt sub-genomes, and the *YAB5*-like group was mainly generated by segmental duplication. The long-terminal repeat retroelements Copia and Gypsy were identified as major transposable elements accompanying the appearance of duplicated YABBY genes, suggesting that transposable element expansion might be involved in gene duplication. Selection pressure analyses using PAML revealed that relaxed purifying selection might be the main impetus during evolution of YABBY genes in the examined species. Furthermore, exon/intron pattern and motif analyses indicated that genes within the same group were significantly conserved between *Arabidopsis* and cotton. In addition, the expression patterns in different tissues suggest that YABBY proteins may play roles in ovule development because YABBYs are highly expressed in ovules. The expression pattern of YABBY genes showed that approximately half of the YABBYs were down-regulated under different stress treatments. Collectively, our results represent a comprehensive genome-wide study of the YABBY gene family, which should be helpful in further detailed studies on the gene function and evolution of YABBY genes in cotton.

## Introduction

The small YABBY gene family is specific to seed plants (Floyd and Bowman, 2007), the members of which encode a class of transcription factors containing two conserved domains: a zinc-finger domain in the N-terminal region, and a YABBY domain (helix-loop-helix motif) in the C-terminal region (Bowman and Eshed, 2000; Kanaya et al., 2002). They have been identified in all seed plants examined to date, and fall within five different classes: CRABS CLAW (CRC), FILAMENTOUS FLOWER (FIL)/YABBY3 (YAB3), INNER NO OUTER (INO), YABBY2 (YAB2), and YABBY5 (YAB5) (Bowman, 2000b; Yamada et al., 2001). YABBY genes play roles in diverse processes such as lamina outgrowth, cell polarity maintenance, and leaf margin establishment (Chen et al., 1999; Siegfried et al., 1999; Watanabe and Okada, 2003; Finet et al., 2016). In *Arabidopsis thaliana*, the following six YABBY genes have been identified: *FILAMENTOUS FLOWER* (*FIL*), *CRABS CLAW* (*CRC*), *INNER NO OUTER* (*INO*), *YAB2, YAB3*, and *YAB5* (Bowman and Smyth, 1999; Lee et al., 2005).

*Arabidopsis FIL* is required for normal flower development and is thought to act redundantly with *YAB2* and *YAB3* because of their overlapping expression patterns and sequence homologies (Chen et al., 1999; Kumaran et al., 1999; Siegfried et al., 1999). In rice, the TOB1-related YABBY genes play roles in flower development similar to their *FIL* orthologs in *Arabidopsis* (Tanaka et al., 2017). The *Arabidopsis CRC* gene is essential for nectary specification and carpel polarity (Alvarez and Smith, 1999), and its ortholog in *Pisum sativum* shows conserved functions in carpel morphogenesis (Fourquin et al., 2014). In rice and maize, homologs in the CRC clade share conserved functions in leaf development by modulating midrib development and regulating plant architecture (Nagasawa et al., 2003; Ohmori et al., 2011; Strable et al., 2017). *Arabidopsis INO* plays an important role in development of the outer integument (Villanueva et al., 1999). The soybean *GmYABBY10* gene functions as a negative regulator in plant resistance to drought (Zhao et al., 2017).

Among the eudicots, *CRC* genes are expressed in abaxial carpels and nectaries (Bowman and Smyth, 1999; Lee et al., 2005), whereas *INO* genes are transcribed in the abaxial outer integument layers (Villanueva et al., 1999). The outer integument and carpels are considered to be typical synapomorphies for angiosperms, and are derived from sporophylls (Yamada et al., 2011). Therefore, the YABBY genes are considered to be significant candidates for participation in evolutionary stem-to-leaf transformation (Floyd and Bowman, 2010; Sarojam et al., 2010). However, the “vegetative YABBY genes” *FIL-like, YAB2-like*, and *YAB5-like* are expressed in the cotyledons, leaves, and floral organs (Golz, 2004; Bartholmes et al., 2012).

The expression patterns of vegetative YABBY genes are associated with several processes, including exclusion from apical meristems and activation of the initiation of lateral organ primordia, and often show asymmetric restriction to the abaxial domain of eudicot primordia (Siegfried et al., 1999; Golz, 2004). Evidence from core eudicots indicates that vegetative YABBY genes act in several aspects of leaf development, and some vegetative YABBY genes also regulate shoot apical meristem development and phyllotaxy (Kumaran et al., 2002; Eshed et al., 2004; Toriba et al., 2007; Sarojam et al., 2010). Thus, the evolutionary history of YABBY genes coincides with the origin of leaves in seed plants (Floyd and Bowman, 2007), and it is necessary to understand the relationship between YABBY genes in different species or within the same species.

Cotton is one of the most important economic crops and is the world's most important source of raw materials for textile fibers and edible oils (Yang et al., 2014; Gong et al., 2017b). Although RNA-seq data for cotton YABBY genes have been presented in a few studies (Jiao et al., 2013; Zhang et al., 2016), further experimental analyses are needed to clarify their biological function. Therefore, it is necessary to understand the biological consequences of YABBY gene family activity in cotton plants. In this regard, completion of the genome sequences of *Gossypium raimondii, Gossypium arboreum*, and *Gossypium hirsutum* and genome-wide analysis of the YABBY gene family have provided a valuable database resource (Wang et al., 2012; Li et al., 2014, 2015; Zhang et al., 2015). Although some YABBY genes have been reported in monocotyledonous and dicotyledonous plants, the relationships between these genes are poorly understood. In this study, we isolated YABBY sequences from eight different species and analyzed the relationship between monocotyledonous and dicotyledonous plants. Furthermore, we focused on cotton, using various methods to analyze YABBY genes, including the identification of gene families, phylogenetic tree analysis, and analyses of segmental duplication, gene structures, chromosome location, and expression patterns.

## Materials and methods

### Sequence identification

The genome sequences of the cotton species *Gossypium arboreum* (BJI, version 1.0), *G. raimondii* (JGI, version 2.0), and *G. hirsutum* (NAU, version 1.1) were downloaded from COTTONGEN (www.cottongen.org) (Yu et al., 2014). The genome sequences of rice (version 7.0), sorghum (version 2.1), maize (version 1.1), cacao (version 1.1), and *A. thaliana* were retrieved from JGI (https://phytozome.jgi.doe.gov/pz/portal.html). The amino acid sequences of YABBYs from *A. thaliana* were acquired from The *Arabidopsis* Information Resource, version 10 (TAIR 10) (http://www.arabidopsis.org). We used YABBY protein sequences from *Arabidopsis* as query sequences to search the *G. arboreum* protein database for candidate sequences, employing the blastp program. We then used Interproscan 63.0 (Jones et al., 2014) to identify the YABBY domain (PF04690) in the candidate sequences. YABBYs in rice, sorghum, maize, cacao, *G. hirsutum*, and *G. raimondii* were analyzed as described above for *G. arboreum*. The blastn program was also used to identify potential un-annotated genes in the cotton genome.

### Conserved sequence and phylogenetic analysis

We used Clustal X 2.0 for multiple-sequence alignments. For conserved domain-sequence analysis, sequence logo analysis of the zinc-finger and YABBY domains from rice, *Arabidopsis*, and upland cotton genes was performed using the online tool WEBLOGO (Crooks et al., 2004) and multiple sequence alignment results were used as the input file. For phylogenetic analysis, the ClustalW program (build-in MEGA 7.0) (Kumar et al., 2016) was used to align the full-length YABBY sequences from rice, sorghum, maize, cacao, *Arabidopsis, G. arboreum, G. raimondii*, and *G. hirsutum*, and a neighbor-joining (NJ) tree was constructed using the bootstrap method with 1000 replicates. Substitution was evaluated with the Poisson model using the default parameters. To validate the NJ tree, the minimum-evolution method was also used. The bootstrap method was used with 1,000 replicates.

### Chromosomal location and collinearity analysis

The exon loci of YABBYs were extracted from the annotation gff3 file using a Perl script. MapChart was used to display gene locations on the chromosome (Voorrips, 2002). For collinearity analysis, the entire YABBY protein sequences of upland cotton were aligned with each other using the Basic Local Alignment Search Tool (BLAST) with a cut-off *e*-value of 1 × 10^−5^. The blastp results were analyzed using MCSCAN software to produce collinearity blocks across the whole genome. Collinear pairs within the YABBY family of proteins were extracted to draw a collinearity map using CIRCOS software (Krzywinski et al., 2009).

### Selective pressure analysis

Amino acid sequences from homologous pairs were aligned using Clustal X 2.0. The alignment results were converted to a codon alignment in PAML style using the PAL2NAL program (Suyama et al., 2006) (http://www.bork.embl.de/pal2nal/). The built-in PAML package of the CODEML program (Yang, 2007) was used to estimate the selective pressure. A branch model and site model were used in the current study. For branch model analysis, two-ratio model 2 and model 0 were used to determine the omega value of each branch. The likelihood ratio test (LRT) was used to assess which was the more suitable of the two models. For site model analysis, the amino acid sequences from each branch were extracted to construct an NJ tree and the corresponding sequences were converted to a codon alignment as an input file for the CONDEML program, as described previously (Cao et al., 2017).

### Annotation and analysis of transposable elements (TEs)

We used *de novo* prediction and a homolog search method based on Repbase (Jurka, 2000) to determine the repeat content of the genomic DNA. Three software programs, namely PILER-DF, RepeatModeler, and LTR_FINDER (Edgar and Myers, 2005; Xu and Wang, 2007), were used to predict TEs in the genome for *de novo* analysis. Furthermore, a known TE library was used to identify TEs at the DNA level with the RepeatMasker program (Chen, 2004), using Repbase TE (Edition-20170127). To analyze the function of TEs in the expansion of the YABBY family, we identified TEs located 10,000 and 2,000 bps upstream and downstream of the YABBY genes and performed statistical analysis of the different types of TEs present.

### Gene structure analysis

The full-length *Arabidopsis* and *G. hirsutum* protein sequences were aligned with ClustalW, and MEGA 7.0 (Kumar et al., 2016) was used to construct an NJ tree using the method and parameters described above. The exon positions were acquired from the gff3 file using a Perl script, and the gene structure was displayed using the online tool GSDS 2.0 (Hu et al., 2014).

### Transcriptome data analysis and gene expression heatmap

The raw RNA-Seq data were downloaded from the NCBI Sequence Read Archive (https://www.ncbi.nlm.nih.gov/bioproject/PRJNA248163/). TopHat (version: 2.0.13) was used for mapping reads, cufflinks (version: 2.2.1) were used to analyze gene expression levels, and fragments per kilobase million values were used to normalize gene expression levels (Trapnell et al., 2013). The expression levels of YABBYs were determined by extracting their respective data from the total expression matrix, using Genesis software (version: 1.7.7) (Sturn et al., 2002).

### Real-time PCR

Upland cotton seeds (CCRI24 variety) were obtained from the Institute of Cotton Research of the Chinese Academy of Agricultural Science. Seed germination was performed on a wet filter paper at 28°C for 3 days, after which the seeds were transferred to liquid culture medium (Yang et al., 2014). At the three-leaf stage, the seedlings were treated with 20% PEG6000. The roots were sampled at 0, 1, 3, and 6 h post-treatment, immediately frozen in liquid nitrogen, and stored at −80°C. In addition, the true leaf, stem, and root under normal conditions were sampled, immediately frozen in liquid nitrogen, and stored at −80°C. Total RNA was purified from the samples using the RNAprep Pure Plant Kit (TIANGEN, Beijing, China), following the manufacturer's recommended protocol. The first strand of cDNA was synthesized using the PrimeScript® RT Reagent Kit (Takara, Dalian, China). Real-time PCR amplifications were performed in an ABI 7900HT instrument (Thermo Fisher, USA) using SYBR Premix Ex Taq™ II (Takara, Dalina, China). Upland cotton histone 3 mRNA (GenBank accession number AF024716) was used as an internal control. The dissociation curves of each reaction were assessed, and the cycle threshold (CT) 2^−ΔΔCT^ method was used to calculate the expression level of each target gene as previously reported (Gong et al., 2017a).

## Results

### Gene identification and homeobox domain retrieval

The sequences of six *Arabidopsis* YABBY genes were obtained from TAIR (http://www.arabidopsis.org) and used as queries for searching the rice, sorghum, maize, cacao, *G. arboreum, G. raimondii*, and *G. hirsutum* databases, using the blastp program with a cut-off *e*-value of 1 × 10^−5^. Thereafter, potential candidates were extracted from the database. InterProscan 63.0 (http://www.ebi.ac.uk/interpro/) was used to search for the YABBY protein (PF04690) among the obtained sequences, and 8, 8, 11, 7, 12, 12, and 23 genes were confirmed as YABBY family members in rice, sorghum, maize, cacao, *G. arboreum, G. raimondii*, and *G. hirsutum*, respectively (Supplementary Table 1). The number of YABBY genes in diploid cotton *G. raimondii* was the same as that in maize, but exceeded that in rice, sorghum and cacao. The number of YABBY genes in upland cotton was almost twice that in the genomes of the two diploid cottons, *G. arboreum* (AA) and *G. raimondii* (DD), in accordance with the hypothesis that upland cotton was derived from hybridization with a progenitor having a diploid genome, after which the relevant chromosomes doubled. *G. raimondii* has a smaller genome size and simpler genome structure compared with that of *G. arboreum* (AA) and *G. hirsutum*, which made genome sequencing and high-quality assembly much easier to accomplish with *G. raimondii* than with *G. arboreum* (AA) and *G. hirsutum*. As shown in Supplementary Table 2, the D genome (*G. raimondii*), Dt-subgenome (*G. hirsutum*), and A genome (*G. arboreum*) contain 12 YABBY genes and the At-genome has 11 genes. Ga, Gr, Gh, and At represent *G. arboreum, G. raimondii, G. hirsutum*, and *A. thaliana*, respectively, and these terms are used as prefixes before the names of the YABBY genes. On the basis of gene number and chromosome locus, we designated YABBY genes from *G. raimondii* as YABBY1 to YABBY12, and genes from the A-genome, At-subgenome, and Dt-subgenome were named after their orthologs in *G. raimondii*. We found that *GhYABBY7*_*Dt* was not available in current cotton databases (Supplementary Table 2), and therefore we used the protein sequence of its ortholog *GhYABBY7_At* as a query to search the upland cotton genomic sequence (Supplementary Table 3). We also extracted the genomic sequence of *GhYABBY7_At* to align with the upland cotton genome. As shown in Supplementary Table 3, a region (from 35585961 to 35588822 bp) on D09 is the potential locus for *GhYABBY7_Dt*. We extracted the genomic sequence around this region to align with the *GhYABBY7_At* genomic sequence and found gaps within this region that disrupted the *GhYABBY7_Dt* sequence (Supplementary Image 1). This region accordingly no longer retains an encoding function, and we consider that the incomplete genomic sequence may explain the absence of *GhYABBY7_Dt* annotation. The orthologs from *G. raimondii*, At-subgenome, and Dt-subgenome are almost all in homologous chromosomes, indicating that they have been conserved during evolution. *GhYABBY11*_*Dt* has 199 amino acid residues, making it larger than the orthologs *GhYABB11*_*At* (183 AA), *GrYABBY11* (183 AA), and *GaYABBY11* (183 AA). Further inspection of the gene structure revealed that *GhYABBY11*_*Dt* has six exons, whereas the other three orthologs have seven exons. Thus, we extracted the genomic sequence of *GhYABBY11*_*Dt*, re-annotated the gene using AUGUSTUS (Stanke et al., 2004), and found the gene has multiple splicing isoforms (Supplementary Table 4), one of which encodes 183 amino acids (similar to *GhYABBY11*_*At* and *GrYABBY11*). We consequently used the novel predicted isoform for subsequent study.

### Conserved amino residues within YABBY proteins

**Figure 1:**
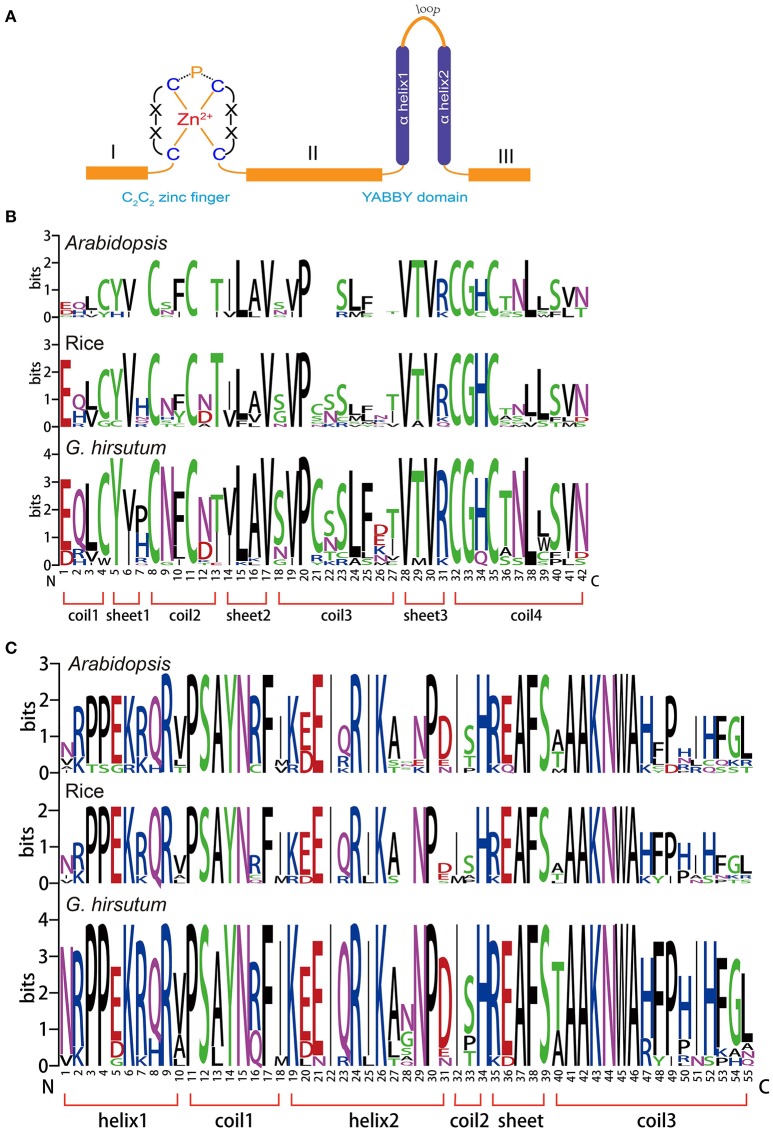
Conserved domains in the YABBY gene family. **(A)** Members of the YABBY gene family are characterized by two highly conserved domains: a C_2_C_2_ zinc-finger domain toward the N terminal and a “YABBY” domain toward the C terminal. **(B)** Sequence logos showing the highly conserved zinc-finger domain. **(C)** Sequence logos showing the highly conserved YABBY domain.

The YABBY gene family is a small family of transcription factors characterized by two conserved domains, a C_2_C_2_ zinc-finger-like domain (located toward the amino terminus) and a YABBY domain (located toward the carboxyl end of the protein), as shown in Figure 1A (Bowman, 2000b). Compared with the conserved domains, we found that domains I, II, and III were more variable in length (Figure 1A). To analyze the homologous domain sequences and the degree of conservation of each residue in the zinc-finger and YABBY domains, multiple sequence alignments were performed to generate sequence logos of both domains in *Arabidopsis*, rice, and *G. hirsutum*. As shown in Figure 1B, we found that both the zinc-finger and YABBY domains are significantly conserved among the three species. Some amino acid residues in the zinc-finger domain were found to be highly conserved, such as L, V, and P in sheet 2; P in coil 3; V, T, and R in sheet 3; and C, G, H, N, and L in coil 4. Most amino acids in the YABBY domain were significantly conserved (Figure 1C).

### Phylogenetic analysis of YABBY genes among typical dicots and monocots

**Figure 2:**
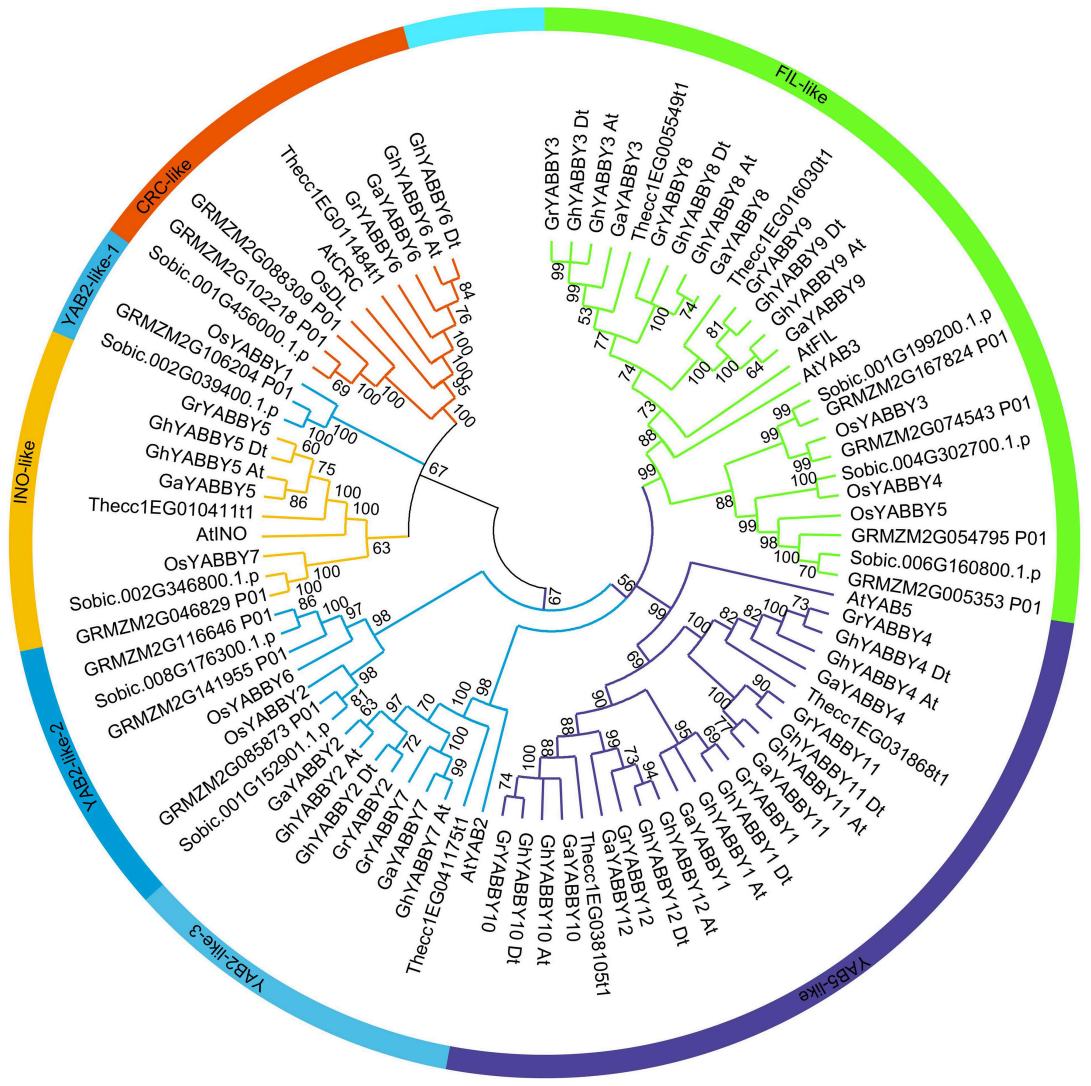
Phylogenetic tree of YABBY genes indicating that YABBY genes can be clustered into five groups. The NJ tree was constructed using MEGA software, version 7.0. The outer circle is marked in orange, yellow, light blue, purple, and green, which represent the *CRC*-like, *INO*-like, *YAB2*-like, *YAB5*-like, and *FIL*-like groups, respectively. The prefixes At, Os, GRMZM, Sobic, Thecc, Ga, Gr, and Gh, represent *Arabidopsis thaliana, Oryza sativa, Zea mays, Sorghum bicolor, Theobroma cacao, Gossypium arboreum, G. raimondii*, and *G. hirsutum*, respectively. “At” and “Dt” indicate the A- and D- sub-genomes in upland cotton, respectively. Bootstrapping was used to test the tree, and only values >50 are displayed near the nodes.

To study the phylogenetic relationship of YABBY genes among cotton plants (*G. hirsutum, G. arboreum*, and *G. raimondii*) and other species, a comprehensive neighbor-joining (NJ) tree was constructed using MEGA 7.0. The phylogenetic tree showed that the YABBY genes can be divided into five groups, named the *CRC*-like, *INO*-like, *YAB2*-like, *YAB5*-like, and *FIL*-like groups, as previously reported (Bartholmes et al., 2012) (Figure 2). To evaluate the accuracy of the NJ tree, a minimum-evolution method was also used to construct a phylogenetic tree (Supplementary Image 2). We found that the topology of the two trees was almost the same, indicating that our tree was suitable for further study. The genes in the *YAB2*-like group can be divided into three sub-groups in the tree. The *FIL*-like group had the most members (26), followed by *YAB5*-like (22), *YAB2*-like (20), *CRC*-like (10), and *INO*-like (9). We found that the *INO*-like group contained one gene at most from each species, indicating that the group has not undergone expansion and that group members may perform similar biological functions. Similarly, with the exception of maize, the *CRC*-like group had only one member from each species. It is noteworthy that the *FIL*-like and *YAB2*-like groups have undergone expansion, although the expansion rates were uneven. In the *YAB2*-like group, the gene number from monocots has increased at least three times, whereas that in the dicots *Arabidopsis* and cacao has not. In contrast, the expansion rate was almost the same between dicots and monocots in the *FIL*-like group. With the exception of the *YAB5*-like group, all other groups comprised both dicot and monocot species (Figure 2), suggesting that genes from monocots in the *YAB5*-like group may have been lost or that this group evolved in dicots after monocots and dicots diverged.

We found that cacao YABBY genes displayed a close relationship with those from cotton (Figure 2). The number of YABBY genes in *G. raimondii* was approximately twice that in cacao, with one cacao gene corresponding to one to three orthologs in cotton. For example, in the *CRC*-like group, *Thecc1EG11484t1* had one ortholog in both *G. arboreum* and *G. raimondii*, whereas in the *FIL*-like group, *Thecc1EG005549t1* had two orthologs in both *G. arboreum* and *G. raimondii*. Furthermore, in the *YAB5*-like group, *Thecc1EG038105t1* had three orthologs in both *G. arboreum* and *G. raimondii*.

As shown in Figure 2 and Supplementary Image 2, most of the orthologs from the A genome and At sub-genome were inclined to cluster together, as was the case with orthologs from the D genome and Dt sub-genome, suggesting that the orthologs from At-A or Dt-D share a close relationship.

### Gene expansion and synteny analysis

**Figure 3:**
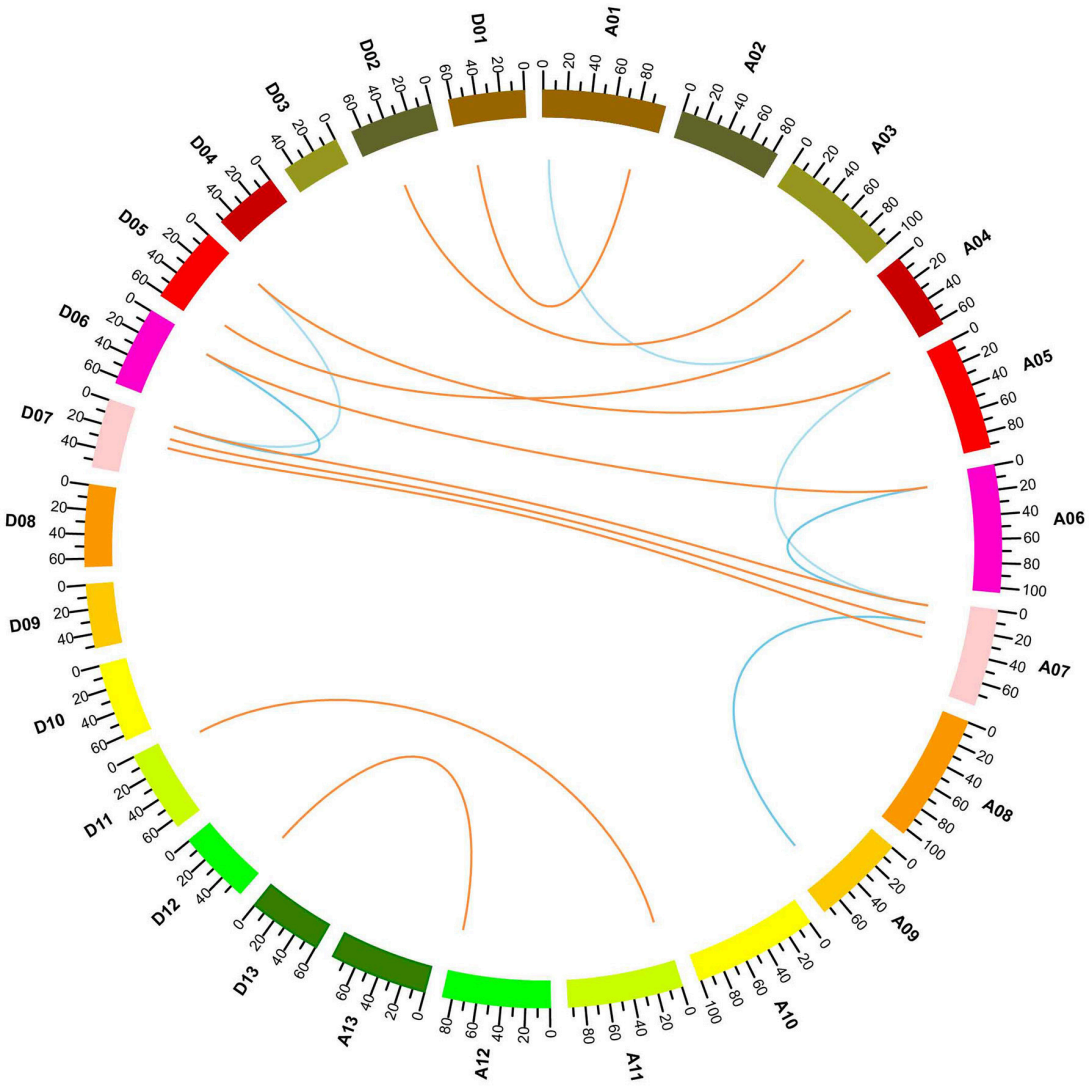
Collinearity analyses within upland cotton. The ends of the orange lines are oriented toward the orthologous genes from the At and Dt sub-genomes. The ends of the blue lines point toward paralog pairs derived from segmental duplication.

**Figure 4:**
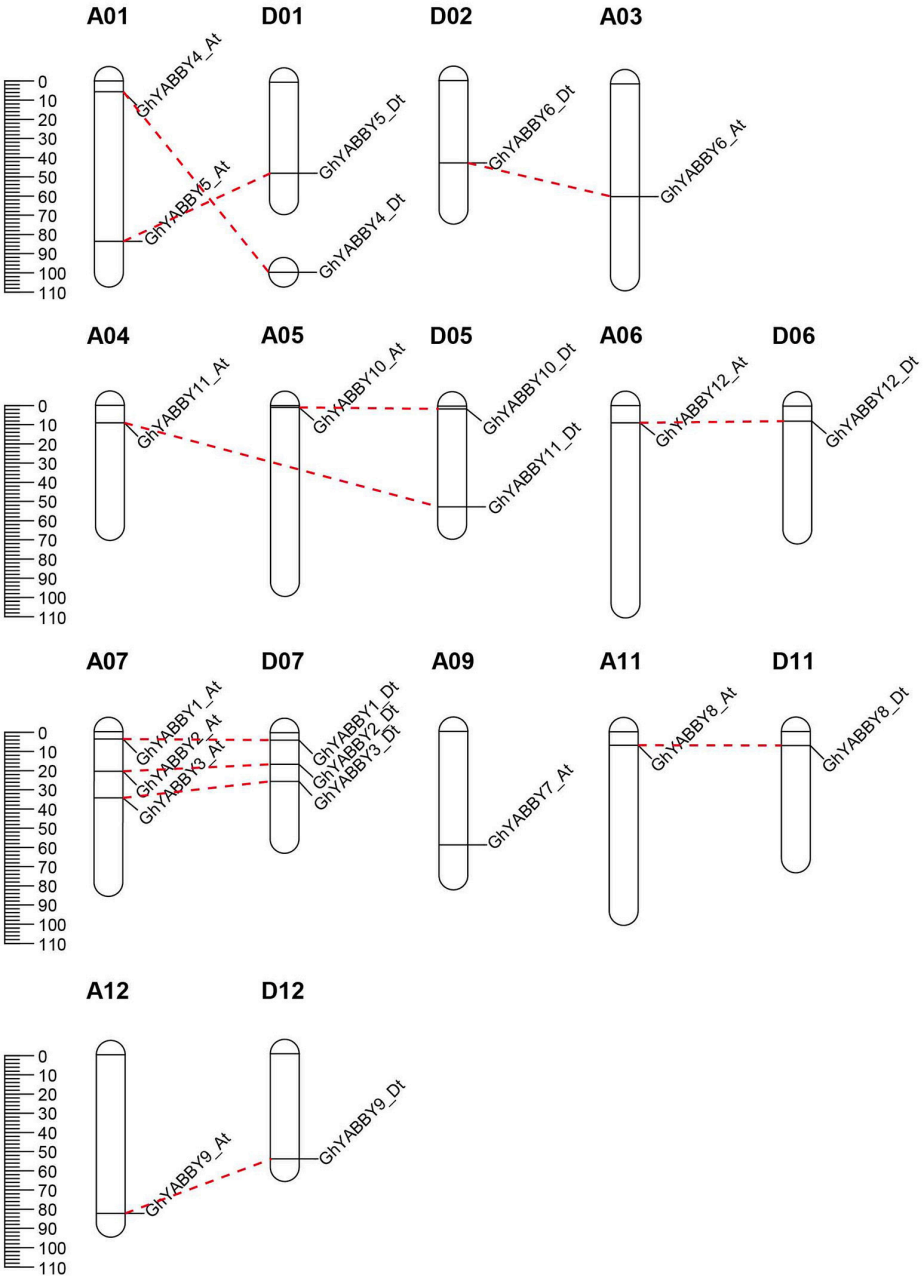
Chromosomal locations of YABBY genes. The red dotted lines link the orthologs located on the At and Dt sub-genomes.

*G. hirsutum* is a typical allotetraploid, which is an ideal material for studying genome polyploidy and its effects (Paterson et al., 2012). To better understand the collinearity relationship of orthologs between the At and Dt genomes, we performed synteny analysis after aligning the gene loci on the chromosomes. The collinearity analysis showed that most of the YABBY loci were significantly conserved between the At and Dt sub-genomes (Figure 3). Twenty-three YABBY genes were distributed unevenly among 16 chromosomes (Figure 4); for example, no YABBY genes were found in chromosomes A02, A08, A13, D03, D04, D08, D09, and D13. We found that nine pairs of orthologs were located on the homologous chromosomes and showed good collinearity. *GhYBBBY4_Dt* was not mapped to a chromosome because of incomplete sequencing. The orthologous pairs *GhYABBY6*_*At*-*GhYABBY6*_*Dt* and *GhYABBY11*_At-*GhYABBY11*_*Dt* were located on non-homologous chromosomes, which might have been caused by chromosome translocation. To validate this possibility, we inspected their orthologs in diploid cotton and found that *GhYABBY6*_*At* and *GaYABBY6* were on homologous chromosomes, as were *GhYABBY6*_*Dt* and *GrYABBY6*, which suggested that chromosomal translocation within the *GhYABBY6* locus occurred before upland cotton speciation. We found that *GhYABBY11*_*Dt* and *GrYABBY11* are located on homologous chromosomes, whereas *GaYBBY11* located on the scaffold.

MCSCAN was used to determine the duplicate gene types. Five types of gene duplication may occur, namely, singleton, dispersed, proximal, tandem, and segmental duplication. Over half of the YABBY genes were identified as singletons, and the remaining 10 out of 23 genes were found to have undergone segmental duplication. Among the duplicated genes, *GhYABB1_At* and *GhYABBY1_Dt* each formed two pairs of collinear genes, suggesting that they were active during evolution. No tandem, proximal duplications (in the nearby chromosomal region, i.e., not in the adjacent region), or dispersed genes were found among the YABBYs (Figure 3 and Supplementary Table 5).

Duplication was the major impetus underlying YABBY gene expansion during evolution. After duplication, the YABBY genes might have undergone functional divergence, and some might have lost their original functions, acquired novel functions, or maintained partition of original functions (Prince and Pickett, 2002; Vandepoele et al., 2003). During the long history of evolution, genes have been exposed to different selective pressures, including positive selection, negative selection, and purifying selection. To better understand the selection pressure among different branches of the phylogenetic tree, the branch model test was used to estimate ω. As shown in Table 1, the *p*-values of the likelihood ratio test (LRT) were smaller than 0.05 in *FIL*-like and *YAB5*-like branches, indicating that the one-ratio model should be rejected. Therefore, the selective pressures associated with the above mentioned two branches were different from those associated with other branches. The mean ω values for *FIL*-like and *YAB5*-like were 0.00038 and 0.01608, respectively, which are smaller than the background values, suggesting that *FIL*-like and *YAB5*-like have been under relaxed purifying selection. Moreover, the mean ω values for the other five branches were also smaller than 1.0, suggesting that they have also been under purifying selection. In addition to the branch model, we also used a site model to examine each clade. As shown in Supplementary Table 6, the ratios of dN/dS (ω) for all the branches under the M0 model were smaller than 1.0, indicating that purifying selection is the major impetus for YABBY evolution. The *p*-values of LRTs for comparison between the M7 model and the M8 model in *FIL*-like and *YAB5*-like clades were smaller than 0.05, indicating that some amino acid sites in these two clades may have undergone positive selection.

**Table 1:** Analysis of patterns of natural selection using PAML.

**Group**	**Model**	**LnL**	**Estimates of parameters**		**LRT**	**ω for branch**
			**Background (ω)**	**Foreground (ω)**	***P*-value**	
FIL-like	Two ratio Model 2	−2710.5	0.1418	0.00038	0.0129	0.00038
	Model 0	−2713.6	0.13531			
YAB5-like	Two ratio Model 2	−2711.7	0.13911	0.01608	0.0478	0.01608
	Model 0	−2713.6	0.13531			
YAB2-like-1	Two ratio Model 2	−2712.9	0.13431	0.54075	0.2463	0.13431
	Model 0	−2713.6	0.13531			
YAB2-like-2	Two ratio Model 2	−2712.4	0.1327	999	0.1181	0.1327
	Model 0	−2713.6	0.13531			
YAB2-like-3	Two ratio Model 2	−2714	0.13513	1.8964	0.3900	0.13513
	Model 0	−2713.6	0.13531			
INO-like	Two ratio Model 2	−2711.9	0.13923	0.00173	0.0605	0.13923
	Model 0	−2713.6	0.13531			
CRC-like	Two ratio Model 2	−2713.2	0.1377	0.00343	0.3459	0.1377
	Model 0	−2713.6	0.13531			

TEs are widely dispersed throughout the whole genome, and the presence of TEs is commonly associated with gene duplication (Oliver et al., 2013). TEs are not only associated with gene expansion but are also associated with the regulation of gene expression in response to stress (Bennetzen and Wang, 2014). To evaluate the relationship between TEs and the YABBY gene family, *de novo* prediction and homology search methods were used to identify TEs in the whole genome, as previously reported (Yang et al., 2017), and we further investigated TEs close to the YABBY genes. As shown in Table 2, analysis of the 2,000 bp region around the YABBY gene locus revealed no DNA transposons, although six retroelements were found, namely, four Copia, one Gypsy, and one Helitron element (Table 2 and Supplementary Table 7). Because only a small number of TEs were detected, we scanned regions up to 10 Kb upstream and downstream around each gene locus, and accordingly succeeded in identifying 48 TEs (one DNA transposon and 47 retroelements). The single DNA transposon was from the MULE-MuDR family, whereas the retroelements mainly contained long-terminal repeats (LTRs), including 29 Copia and 17 Gypsy retroelements (Table 2 and Supplementary Table 8). We further inspected the TE distribution and found one Gypsy TE located downstream of *GhYABBY4*_*At* and two Copia TEs located downstream of *GhYABBY12*_*At* within a 2,000 bp region. Within the 10,000 bp region, we found TEs located in the vicinity of 13 gene loci, among which 33 were located around genes showing segmental duplication. For example, four Copia TEs were located downstream of *GhYABBY12*_*Dt*, 11 Copia TEs were located upstream or downstream of *GhYABBY12*_*At*, five Gypsy TEs were located upstream or downstream of *GhYABBY1*_*At*, and two Gypsy TEs were located upstream of *GhYABBY1*_*Dt*. As reported previously, the occurrence of most TEs was correlated with the presence of duplicated genes in the WOX family (Yang et al., 2017). Consistently, we found that this general rule applied to the YABBY gene family, indicating that retroelements (particularly LTRs) may have played significant roles in YABBY family expansion. In contrast to TEs, simple repeat sequences are more abundant and variable, and are distributed widely in the upstream, downstream, and intragenic regions of YABBY genes.

**Table 2:** Transporsable elements around the YABBY gene loci.

**Type**		**Elements number**	**Length occupied**	**Percentage of sequence (%)**	**Elements number**	**Length occupied**	**Percentage of sequence (%)**
		**2,000-bp region**			**10,000-bp region**		
DNA transponsons		0	0 bp	0	1	57	0.01
	CMC-EnSpm	0	0 bp	0	0	0	0
	MULE-MuDR	0	0 bp	0	1	57	0.01
	PIF-Harbinger	0	0 bp	0	0	0	0
	TcMar-Pogo	0	0 bp	0	0	0	0
	hAT	0	0 bp	0	0	0	0
	hAT-Ac	0	0 bp	0	0	0	0
	hAT-Tag1	0	0 bp	0	0	0	0
	hAT-Tip100	0	0 bp	0	0	0	0
	hAT-Charlie	0	0 bp	0	0	0	0
Retroelements		6	1,849	1.21	47	18,477	3.66
	LINE:	0	0	0	0	0	0
	L1	0	0	0	0	0	0
	LTR:	5	1,794	1.17	46	18,422	3.65
	Caulimovirus	0	0	0	0	0	0
	Copia	4	1,731	1.13	29	12,604	2.5
	Gypsy	1	63	0.04	17	5,818	1.15
	RC:	1	55	0.04	1	55	0.01
	Helitron	1	55	0.04	1	55	0.01
Low_complexity		22	1,030	0.67	74	3,846	0.76
Simple_repeat		107	3,938	2.58	294	12,377	2.45
Unspecified		3	541	0.35	70	29,361	5.82

### Gene structure and motif analysis

**Figure 5:**
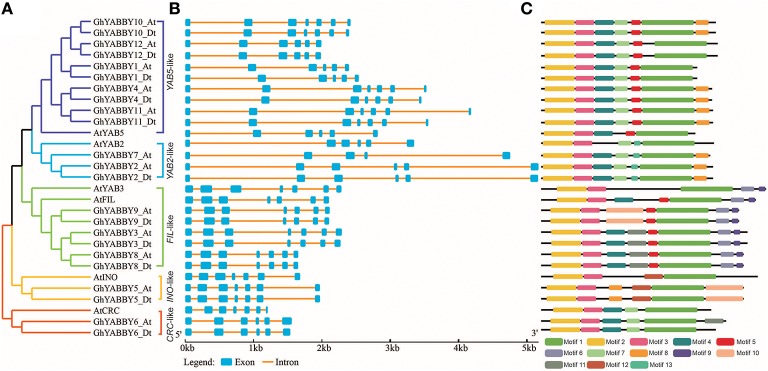
Comparison of the gene structures between *Arabidopsis thaliana* and *Gossypium hirsutum*. **(A)** NJ tree analysis of *A. thaliana* and *G. hirsutum*. Orange, yellow, light blue, purple, and green represent the *CRC*-like, *INO*-like, *YAB2*-like, *YAB5*-like, and *FIL*-like groups, respectively. **(B)** The number, length, and position of exons and introns within YABBY genes. Boxes indicate the exons and black lines indicate the introns. **(C)** Distribution of the predicted motifs in the YABBY genes.

To further study the phylogenetic relationship among YABBYs from upland cotton and *Arabidopsis*, we compared their gene structures and motifs. As shown in Figure 5A, the YABBYs were divided into the five groups described in Figure 2. Because the number of genes used for constructing the phylogenetic trees shown in Figures 2, 5A were different, the resulting topologies of these trees also differed. However, the gene members and topologies within groups were almost the same. As is evident from Figure 5, YABBYs in the *YAB5*-like group might have undergone expansion because one *AtYAB5* gene corresponds to 10 homologous genes from upland cotton (Figure 5A), and this hypothesis was confirmed through synteny analysis with the At and Dt sub-genomes from upland cotton (Figure 3). As shown in Figure 5B, YABBYs are multiple-exon genes (five to seven exons), and the genes close to each other in the phylogenetic tree showed highly similar exon patterns. For example, all YABBY genes in the *FIL*-like and *INO*-like groups have seven exons, most YABBY genes in the *YAB2*-like group have six exons, and most in the *YAB5*-like group have seven exons. Comparing exons in orthologs from the same position, we found that the exon sequences were more conserved than the intron sequences. For example, the first intron sequence of *GhYABBY11*_*Dt* was longer than that of *GhYABBY11*_*At*, and the length of the sixth intron of *GhYABB10*_*Dt* was shorter than that of *GhYABB10*_At. We found that the length of the sixth exons of *GhYABBY6*_At/Dt were not equal, which may have been caused by a premature stop codon. Indeed, further analysis of the genomic sequences of *GhYABBY6*_*At*/*Dt* revealed a 4 bp indel in the sixth exon of *GhYABBY6*_*Dt*, resulting in a frameshift mutation and a premature stop codon (Supplementary Image 3).

MEME was used to discover the possible motifs within YABBY genes to better understand their phylogenetic relationships. A total of 13 motifs were identified in the YABBY genes, the widths of which ranged from 8 to 57 amino acids. Motif 1 is located in the YABBY domain, and motifs 2 and 3 are located in the C2C2 zinc-finger-like domain (Supplementary Table 9 and Figure 5C). As shown in Figure 5C, in addition to the common motifs 1, 2, and 3 harbored by all the YABBY genes analyzed, some private motifs were only found in genes within the same group. For example, all genes within the *YAB5*-like group were characterized by motifs 4 (QS[LH]S[GW][QH][DS][IF][QF][AT]P[NQ][YN][AT]L[ES][ED]YRSD) and 5 ([NE][ETN][IAT][TP][EKQ][EP][RP]VVNR), whereas genes in the *FIL*-like group contained motifs 5 ([NE][ETN][IAT][TP][EKQ][EP][RP]VVNR) and 6 ([RH]QQEGE[ED][MAV][VL][MV]KDGFF).

### Gene expression patterns in different tissues under multiple stresses

**Figure 6:**
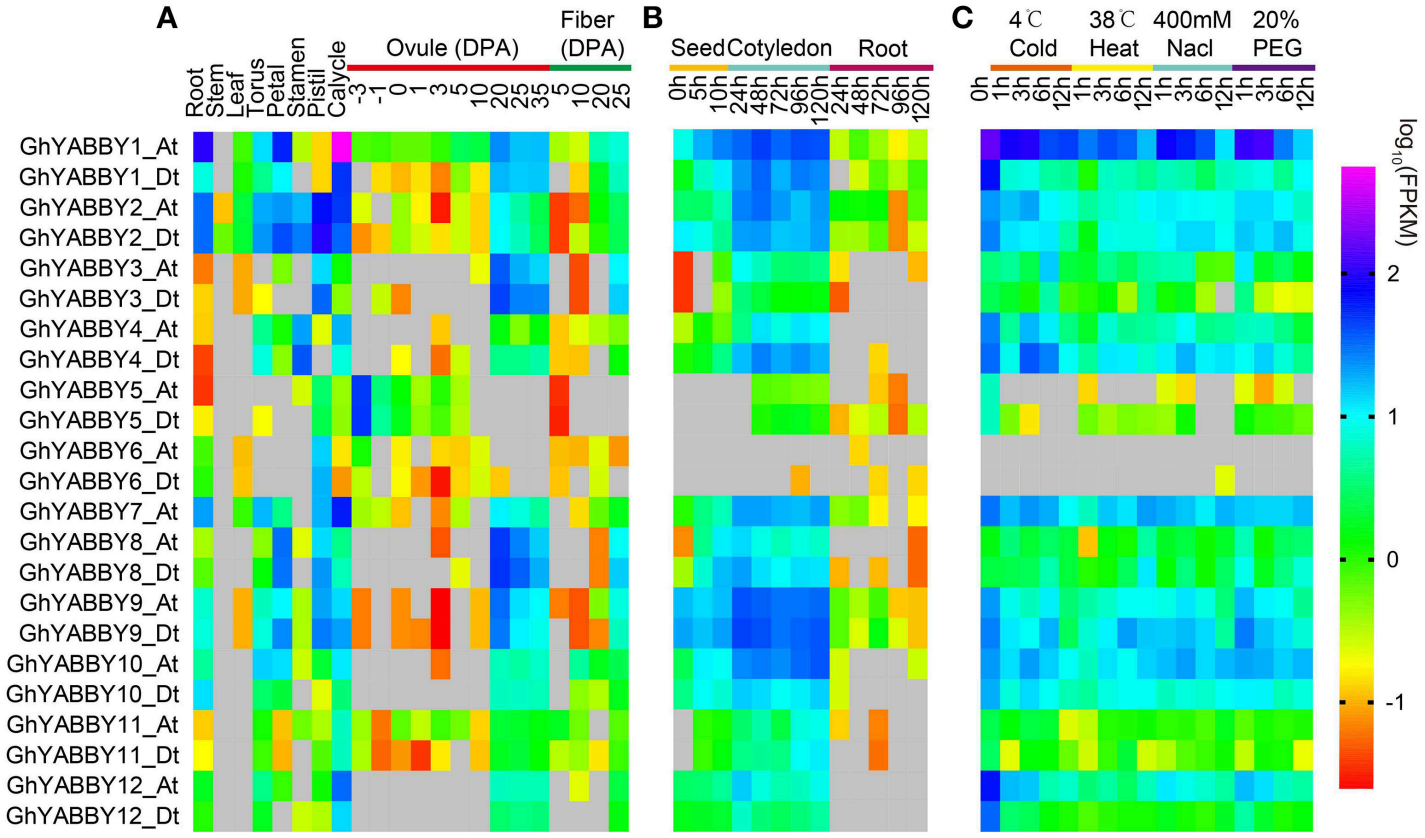
Gene expression profiles of YABBY genes in different tissues **(A)**, during seed germination **(B)**, and under different stresses **(C)**. True leaves were harvested at 0, 1, 3, 6, and 12 h after treatment.

Gene expression is the major step toward a gene realizing its biological function. Thus, we investigated YABBY gene expression profiles in different tissues, during seed germination and seed (fiber) development, and under biological stresses. RNA-Seq data were acquired and analyzed as described in our previous report (Yang et al., 2017). As shown in Figure 6A, YABBY genes exhibited different expression patterns in differing vegetative tissues (roots, stems, and leaves), reproductive tissues (torus, petal, stamen, pistil, and calycle), stages of seed development (−3, −1, 0, 1, 3, 5, 10, 20, 25, and 35 days post-anthesis [DPA] ovule), and stages of fiber development (5, 10, 20, and 25 DPA), suggesting that YABBYs have multiple biological functions. With the exception of *GhYABBY2_At/Dt*, the expression of other genes was not detectable in the stem. The YABBY genes have a relative low expression level in leaves. All the genes were transcribed in roots and calycles. Most genes were highly expressed in the late stage of ovule development (20–35 DPA) and 25 DPA fibers (Figure 6A), indicating that YABBYs might play important roles in seed development and secondary wall development, suggesting that these are potential candidate genes for further study. However, *GhYABB5_At/Dt* were highly expressed during the early stages of ovule development (−3 DPA and−1 DPA). *GhYABBY6*_*At*/*Dt* have a relatively low expression level during ovule development, but were preferentially expressed in the pistil (carpel). YABBYs might be associated with seed germination, considering that almost all these genes were highly expressed in cotyledons at different sampling stages (Figure 6B). When comparing the expression profiles of the orthologs between the At and Dt sub-genomes, the expression patterns and levels of the orthologous pairs were similar, suggesting that their biological functions might be conserved. When comparing the expression patterns of homologous pairs, we found that *GhYABB1*_*At*/*Dt* were expressed at all the stage of ovule development, showing a down-regulated pattern, whereas *GhYABBY10*_*At*/*Dt* and *GhYABBY12*_*At*/*Dt* were transcribed in the late stage of ovule development, suggesting that functional divergence might have occurred during evolution after duplication.

Cotton is the most important cash crop for fiber production; however, it faces multiple stresses during growth and development. Therefore, we performed a comprehensive study of the expression pattern of YABBYs in this study. When cotton was exposed to cold treatment, most genes displayed a down-regulated expression pattern, i.e., the expression levels of *GhYABBY1_Dt, GhYABBY2_At/Dt, GhYABBY4_At, GhYABBY9_At/Dt, GhYABBY10_At/Dt*, and *GhYABBY12_At/Dt* were down-regulated and when exposed to cold treatment. We found that only *GhYABBY3*_*At*/*Dt* and *GhYABBY8*_*At*/*Dt* from the same group were up-regulated under cold stress. When cotton was exposed to high temperatures, approximately half of the YABBY genes showed a down-regulated pattern, including *GhYABBY1_Dt, GhYABBY2_At/Dt*, and *GhYABBY4_At/Dt*. Similar to the patterns under low and high temperature treatments, approximately half of the YABBY genes displayed down-regulated patterns under PEG and NaCl treatments, indicating that YABBYs might be negative regulators under exposure to stress.

### Evaluation of YABBY gene expression by quantitative PCR (qPCR)

**Figure 7:**
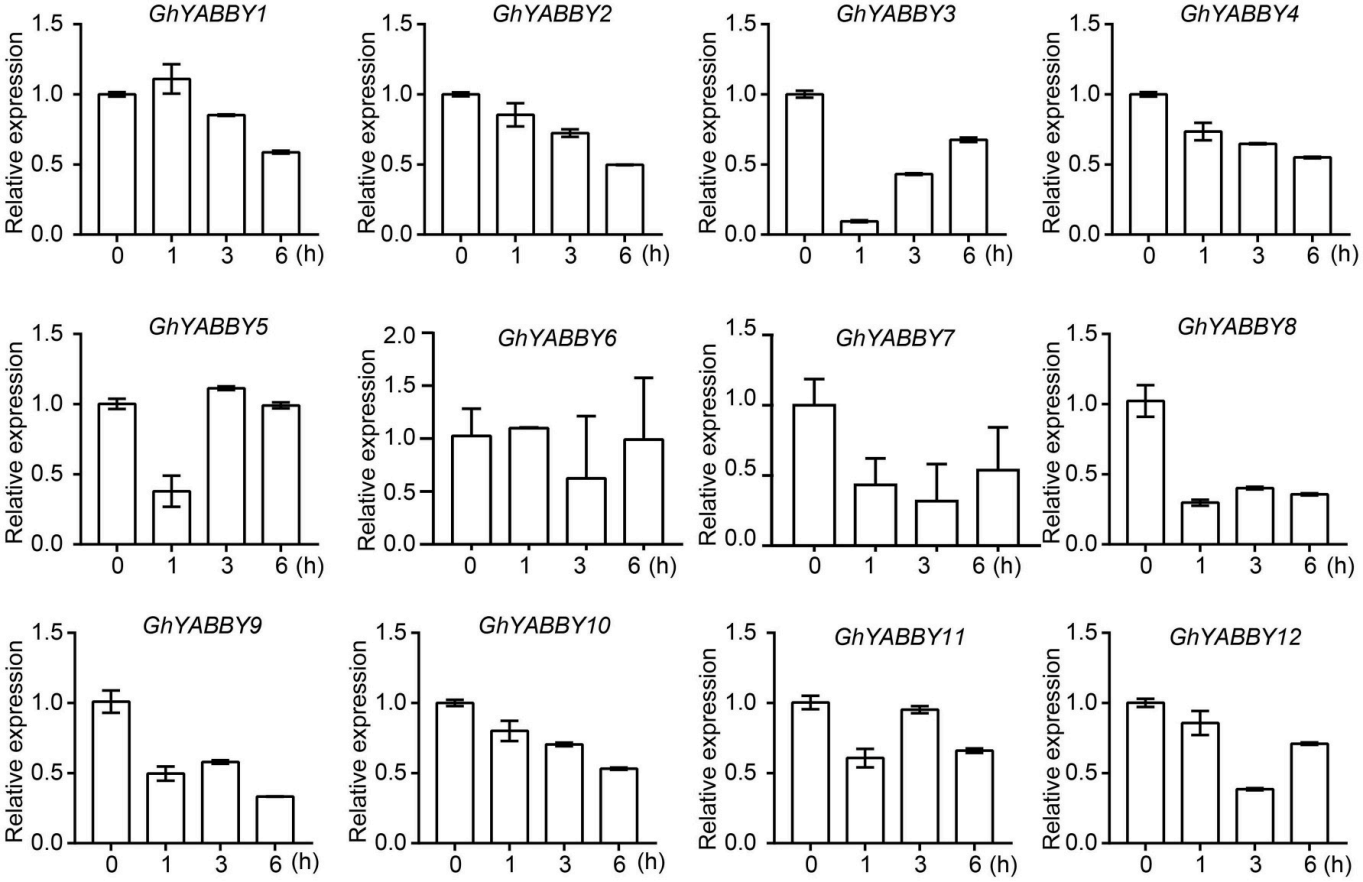
Expression levels of YABBY genes under PEG6000 treatment. Error bars represent the standard deviation of three independent experiments.

To validate and further characterize the YABBY gene expression profiles from the RNA-Seq data, qPCR was performed using root samples treated with PEG6000. Root samples were used because of their relatively higher gene expression levels in most samples. Given that orthologs from the At and Dt sub-genomes have highly similar mRNA sequences, it is difficult to distinguish such orthologous pairs by qPCR. Therefore, primers were designed to amplify the orthologous pairs together (Supplementary Table 10). Figure 6C shows that *GhYABBY6_At/Dt* were not detectable in response to PEG treatment, and we found that *GhYABBY6*_*At*/*Dt* were transcribed at a relatively low expression level since the cycle threshold (*Ct*) values were larger than 30 for all samples, whereas the *Ct* values for the internal control gene (histone 3) were in the normal range (17–18) for the same samples. As shown in Figure 7, most genes were down-regulated following PEG6000 exposure, which is similar to the expression pattern shown in Figure 6C, indicating that our RNA-Seq data were relatively accurate. The expression levels of *GhYABBY2, GhYABBY3, GhYABBY8, GhYABBY9*, and *GhYABBY10* were down-regulated under PEG6000 treatment, suggesting that they may function as negative regulators under drought stress and are potential candidate genes for further study.

## Discussion

YABBY genes are seed plant-specific transcription factors that are associated with leaf, shoot, and flower development. They have been well studied in *A. thaliana*, tomato, and rice (Siegfried et al., 1999; Toriba et al., 2007; Bartholmes et al., 2012; Huang et al., 2013). However, to date, few studies have been performed on YABBY genes in cotton. In the current study, we performed comprehensive identification and analysis of YABBY genes in cotton, with the aim of gaining a better understanding of their functional roles in further studies.

### Duplication of YABBY genes in cotton

Upland cotton is a typical allotetraploid and is an ideal plant for studying polyploid formation in evolution (Paterson et al., 2012). Previous findings have suggested that diploid species resembling *G. arboreum* (A2) and *G. raimondii* (D5) hybridized naturally, followed by chromosome doubling, and natural and human selection, with the eventual formation of upland cotton. In our study, we found that the number of YABBY genes in upland cotton is close to the total number in *G. arboreum* (A2) and *G. raimondii* (D5), suggesting that whole genome duplication was the major impetus underlying the expansion of YABBY genes in upland cotton. Polyploidization has previously been identified in more than 90% of flowering plants, and has played important roles in the adaptation of plants to the environment (Ramsey et al., 1998). Polyploidization has resulted in the enhancement of gene dosages and has led to the strengthening of gene expression, which might have enhanced the yield, fiber quality, biomass, and stress tolerance of the allotetraploids *G. hirsutum* and *G. barbadense* compared with *G. raimondii*, and *G. arboreum*.

As described in a previous study (Fraser et al., 2005), major chromosome events such as segmental duplication and translocation have enabled plants to rapidly adapt to new environments. In the current study, we found that the YABBYs from cotton were well divided into five groups, as observed previously (Bartholmes et al., 2012), and no additional groups were identified in cotton. Among the five groups, the *YAB5*-like group tended to retain recently duplicated paralogs in cotton (Figure 2). Five duplicated gene pairs were found in the *YAB5*-like group and they displayed different expression patterns, indicating that their biological functions might have diverged after duplication and that these genes might have helped upland cotton adapt to new environments. We also found that WOX genes in upland cotton have undergone segmental duplication and that the duplicated genes displayed different expression patterns (Yang et al., 2017), suggesting that divergence is a common evolutionary process after duplication. It appears that the *YAB5*-like group is dicot-specific because all members within this group are dicots. Whether monocots were lost from this group or dicots acquired a specific genetic requirement necessitates further study. Homologs in the *YAB5*-like group appear to have been active during evolution because some duplication events were found in this group. Although the function of *AtYAB5* has yet to be determined, the functions of homologs in cotton from the same group may have diverged because their expression patterns were different, although elucidating their functions requires further study.

Tandem duplication has commonly been found within plant genomes and is a basic contributor to gene family expansion. Unequal chromosome crossover events, chromosomal anomalies, transposon insertions, and other reverse transcriptase-mediated processes can result in tandem duplication, and the new genes would co-exist with the homolog(s) already present (Flagel and Wendel, 2009). One example of tandem duplication is seen in the NBS-LRR gene family, which functions as the major class of disease resistance genes in flowering plants. Among the 166 NBS-LRR genes, 40 clusters have previously been identified (Leister, 2004). Functional redundancy has been considered an important impetus for plant environmental adaption. Neofunctionalization, duplication, degeneration, complementation, subfunctionalization, escape from adaptive conflict, and dosage-balance models have been proposed to help understand gene-duplication events (Flagel and Wendel, 2009).

In addition to gene expansion, gene loss has also occurred during the evolution of upland cotton. The occurrence of gene loss has been accompanied by “genome shock,” such as the rapid arrangement of genomic sequences during hybridization and following chromosome doubling during polyploidization (Paterson et al., 2004).

Transposable elements (TEs) are mobile genetic elements that are located at varying positions in genomic DNA using element-encoded enzymatic machinery (Arkhipova, 2017). Mobile element activity together with recombination are the major processes promoting genome divergence, which plays a key role in the evolution of species. TEs are divided into two classes according to whether TE mobility requires an RNA intermediate (Class I, or retrotransposons) or not (Class II, or DNA transposons) (Finnegan, 1989). Retrotransposons are the most common TEs in genomic sequences, and can be divided into long terminal repeat retrotransposons (LTR) and non-LTR type. In our study, we analyzed the TEs around YABBY gene loci and found that most of the identified TEs were retrotransposons, which is consistent with the pattern in *Helianthus* species (Mascagni et al., 2017). In upland cotton, TEs account for at least 64.8% of the assembled genome, and Gypsy and Copia were the most abundant types in the assembled genome. Although we only analyzed TEs around the YABBY loci, the local TEs display a pattern similar to that detected in genome-wide analysis, which indicated that the identified TEs were mainly of the Gypsy and Copia types. Zhang et al. considered that TEs occurred in the progenitor genomes and were retained after allopolyploid formation (Zhang et al., 2015). In our study, we found the same types of TEs around *GhYABBY1_At/Dt* and *GhYABBY12_At/Dt*, suggesting that these TEs are ancient TEs that occurred in the progenitor genomes of upland cotton.

### Conservation of cotton YABBYs during evolution

YABBY genes are seed plant-specific transcription regulators, characterized by an N-terminal zinc-finger domain and a C-terminal YABBY domain (Figure 1A). Previous studies have shown that the C-X_2_-C-X_9_-P-X_11_-C-X_2_-C motif is a core element forming the C_2_C_2_-type zinc-finger with Zn^2+^, and this structure has also been found in cotton (Leister, 2004; Toriba et al., 2007), indicating that C_2_C_2_-type zinc-fingers are conserved among different species. The putative helix-loop-helix “YABBY” domain has been reported to share sequence similarity with the first two helices of the high mobility group (HMG) box (Leister, 2004), and we found that cotton YABBYs also contain helices typical of HMG boxes, suggesting that YABBYs are conserved among seed plants. Although there is variation in the length of sequences in the internal region between the zinc-finger and YABBY domains, C-terminal region, and N-terminal region, no amino acid insertions/deletions were identified in the aforementioned two domains, and amino acids identities in the YABBY domains were higher than those in the zinc-finger domains, which is consistent with previous findings (Siegfried et al., 1999).

The YABBY genes from Arabidopsis and rice can be divided into five groups: the *INO*-like, *YAB2*-like, *YAB5*-like, *FIL*-like, and *CRC*-like groups (Finet et al., 2016). Twenty-three genes from upland cotton were well divided into these five groups (Figures 2, 4). Genes within the same groups displayed similar characteristics. Firstly, the gene sequences showed higher similarities among genes from the same group. Secondly, apart from the conserved domain regions, the other regions also showed similarities, as evidenced by the finding that different motifs from genes within the same group were more similar (Figure 3C), although they were different within the whole family. Thirdly, exon numbers within the same group were conserved. Our results indicated that genes within the same clade diverged from a common ancestor and may have similar biological functions, suggesting interesting topics for further study. Cacao and cotton plants, which belong to the Malvaceae family, share a common ancestor, and were shown to have a close relationship in the phylogenetic tree (Figure 2). However, the gene numbers in diploid cotton are considerably larger than those in cacao. Previous data have shown that diploid cotton and cacao plants underwent a common ancient duplication event, and that diploid cotton experienced a recent independent duplication event (Li et al., 2014). Therefore, the theoretical number of diploid cotton genes should be twice that of cacao, and a single ortholog in cacao should correspond to two in cotton. However, not all the orthologs in cacao have two orthologs in cotton. We speculate that some cotton genes have been lost or mutated to pseudogenes after recent duplication events during evolution, which has also been found in the HD-ZIP and MIKC MADS-box gene families (Chen et al., 2017; Ren et al., 2017).

### Diverse expression patterns of YABBYs

Cotton is the most important cash crop for fiber and seed oil production, providing raw materials for the textile industry. Cotton fiber cells originate from the epidermis of the outer integument of ovules. In flowering plants, seeds are the most important reproductive organ formed from ovules. Angiosperm ovules have one or two integuments covering the nucellus and female gametophyte, and the integuments develop into a seed coat after fertilization. Numerous studies have focused on ovule ontogeny in *Arabidopsis* (Robinson-Beers et al., 1992; Schneitz, 1999), and many genes associated with ovule development have been identified (Skinner et al., 2004), among which genes involved in cell polarity have been demonstrated to play key roles in ovule development (Villanueva et al., 1999; Eshed et al., 2001; McAbee et al., 2006).

Kelley et al. showed that abaxial determinants are necessary for the initiation and maintenance of integument growth and that the KANADI and YABBY gene families are the two major putative polarity determinants involved in integument development (Kelley et al., 2009). Members of the KANADI gene family control cell polarity and play important roles in integument development. ABERRANT TESTA SHAPE (*ATS*, also known as *KAN4*), a member of the KANADI gene family, is essential for laminar extension during inner integument development and for maintaining integument separation (McAbee et al., 2006). Other KANADI members (*KAN1* and *KAN2*) were found to be involved in outer integument development and are necessary regulators for laminar extension of the outer integument. YABBY genes, a small plant-specific gene family, were previously certified to play roles in abaxial cell fate specification (Bowman, 2000b). *INO*, a member of the YABBY gene family, together with *KAN1* and *KAN2*, were found to play significant roles in polar differentiation of the outer integument (Simon et al., 2012). In a previous study, YABBY2 and YABBY3 were shown to be expressed in a polar manner in all lateral organ primordia and were proposed as key regulators responsible for the specification of abaxial cell fate in *Arabidopsis* (Sawa et al., 1999; Siegfried et al., 1999). CRABS CLAW (CRC) expression is restricted to nectaries and carpels. Although loss of CRC function does not result in any change in carpel polarity, a double mutant of CRC and either PICKLE or KANADI was found to exhibit abaxial abnormity (Bowman, 2000a). In addition, CRC has been reported to play derived roles in leaf vascular development, and an ancestral role of *CRC* orthologs has been implicated in carpel development in the Poaceae family (Fourquin et al., 2014). In the current study, we found that most YABBY genes were expressed at relatively high levels in 25–35 DPA ovules and in early-stage seed germination, which indicates that the functions of YABBYs are conserved between cotton and *Arabidopsis*, and play important roles in ovule development. In contrast, only *GhYBBY5*_*At*/*Dt* were specifically highly expressed during an early stage of ovule development, which indicates that *GhYABB5*_*At*/*Dt* play important roles in early-stage ovule development. Although YABBYs have undergone expansion in cotton, *GhYABBY5* was the only ortholog of *INO* in cotton, and therefore *GhYABBY5*_*At*/*Dt* may share a similar function with *INO* in outer integument differentiation during the early stage of ovule development and are candidate genes for studying cotton ovule development. *GhYABBY6*_*At*/*Dt* are preferentially transcribed in the pistil (carpel), and their expression patterns are similar to those in *Arabidopsis* and Poaceae taxa (Bowman, 2000a; Fourquin et al., 2014), indicating that *CRC* and its orthologs shared conserved functions during evolution after speciation.

The establishment of polarity is a critical process in the development of nearly all lateral organs, and is also required for lamina expansion. Previous data showed that a juxtaposition of abaxial and abaxial domains is essential for lamina development (Waites and Hudson, 1995). YABBYs and KANADIs are the two major gene families required for promoting abaxial cell fate and have been proposed to play important roles in asymmetric leaf development and blade expansion in *Arabidopsis* (Eshed et al., 2004). In the current study, only five gene pairs were detectable in leaves with a relatively high expression level by RNA-seq, among which *GhYABBY2*_*At*/*Dt* and *GhYABBY7*_*At* were close to each other in the phylogenetic tree and are orthologs of *AtYAB2*, which is a gene that is essential for determining lamina outgrowth (Siegfried et al., 1999). Therefore, *GhYABBY2*_*At*/*Dt* and *GhYABBY7*_*At* may participate in lamina development. Compared with other tissues, YABBY genes were almost undetectable in stems, suggesting that YABBYs play functional roles in restricted regions.

YABBYs are associated with the growth and development of lateral organs under both normal growth and stressed conditions. Peng et al. found that YABBY genes were upregulated under cold stress and proposed that transcription factors of the YABBY family and other families, by interacting with Auxin and other hormones, were essential for regulating growth and development of paper mulberry under cold stress (Peng et al., 2015). In our study, only *GhYABBY3*_*At*/*Dt* and *GhYABBY8*_*At*/*Dt* were up-regulated under cold treatment, indicating that these genes may be key regulators of lateral organ development under cold stress. Moumeni et al. found that drought-responsive genes were characterized by the presence of a GCAC[AG][ACGT][AT]TCCC[AG]A[ACGT]G[CT] motif in the promoter region, which is common to AP1, AP2, HD-ZIP, and YABBY, indicating that these transcription factors may play roles in drought response (Moumeni et al., 2015). OsYABBY1 in rice might be associated with leaf rolling during drought stress (Dai et al., 2007). In our study, we found that most YABBY genes were down-regulated under drought and salinity stress, indicating that they might negatively regulate drought or salinity responses. Under heat stress, both up- and down-regulated YABBY genes were detected in our study, indicating that they may serve diverse functions under heat stress. Although the RNA-Seq data revealed the expression pattern of YABBYs under different stress conditions, further studies are needed to unravel their functions.

## Author contributions

FL and ZhY: Conceived and designed the study; YJ, JX, LW, ZL, WQ, and ZuY: Performed the experiments; QG: Prepared the figures; LL: Analyzed and interpreted the data; ZhY, QG, and FL: prepared the manuscript; QC: participated in the design of the experiments, wrote part of the manuscript, and performed a critical review for intellectual content. All authors have read, edited, and approved the current version of the manuscript.

### Conflict of interest statement

The authors declare that the research was conducted in the absence of any commercial or financial relationships that could be construed as a potential conflict of interest. The reviewer DH and handling Editor declared their shared affiliation.
