# Supplementary material for: Genome-Wide Study of YABBY Genes in Upland Cotton and Their Expression Patterns under Different Stresses
Source: Front Genet. 2018 Feb 7;9:33. doi: 10.3389/fgene.2018.00033 (PMC5808293; doi:10.3389/fgene.2018.00033)
Supplement: Supplementary file 1 [file Table1.DOCX]

**Supplementary Table 1. Protein sequences used in this study**

>AtYAB2

MSVDFSSERVCYVHCSFCTTILAVSVPYASLFTLVTVRCGHCTNLLSLNIGVSLHQTSAP

PIHQDLQPHRQHTTSLVTRKDCASSSRSTNNLSENIDREAPRMPPIRPPEKRQRVPSAYN

RFIKEEIQRIKACNPEISHREAFSTAAKNWAHFPHIHFGLKLDGNKKGKQLDQSVAGQKS

NGYY

>AtINO

MTKLPNMTTTLNHLFDLPGQICHVQCGFCTTILLVSVPFTSLSMVVTVRCGHCTSLLSVN

LMKASFIPLHLLASLSHLDETGKEEVAATDGVEEEAWKVNQEKENSPTTLVSSSDNEDED

VSRVYQVVNKPPEKRQRAPSAYNCFIKEEIRRLKAQNPSMAHKEAFSLAAKNWAHFPPAH

NKRAASDQCFCEEDNNAILPCNVFEDHEESNNGFRERKAQRHSIWGKSPFE

>AtCRC

MNLEEKPTMTASRASPQAEHLYYVRCSICNTILAVGIPLKRMLDTVTVKCGHCGNLSFLT

TTPPLQGHVSLTLQMQSFGGSDYKKGSSSSSSSSTSSDQPPSPSPPFVVKPPEKKQRLPS

AYNRFMRDEIQRIKSANPEIPHREAFSAAAKNWAKYIPNSPTSITSGGHNMIHGLGFGEK

K

>AtYAB5

MANSVMATEQLCYIPCNFCNIILAVNVPCSSLFDIVTVRCGHCTNLWSVNMAAALQSLSR

PNFQATNYAVPEYGSSSRSHTKIPSRISTRTITEQRIVNRPPEKRQRVPSAYNQFIKEEI

QRIKANNPDISHREAFSTAAKNWAHFPHIHFGLMLESNKQAKIA

>AtFIL

MSMSSMSSPSSAVCSPDHFSPSDHLCYVQCNFCQTILAVNVPYTSLFKTVTVRCGCCTNL

LSVNMRSYVLPASNQLQLQLGPHSYFNPQDILEELRDAPSNMNMMMMNQHPTMNDIPSFM

DLHQQHEIPKAPPVNRPPEKRQRVPSAYNRFIKEEIQRIKAGNPDISHREAFSAAAKNWA

HFPHIHFGLVPDNQPVKKTNMPQQEGEDNMVMKEGFYAPAAANVGVTPY

>AtYAB3

MSSMSMSSSSAPAFPPDHFSSTDQLCYVHCSFCDTVLAVSVPPSSLFKTVTVRCGHCSNL

LSVTVSMRALLLPSVSNLGHSFLPPPPPPPPPNLLEEMRSGGQNINMNMMMSHHASAHHP

NEHLVMATRNGRSVDHLQEMPRPPPANRPPEKRQRVPSAYNRFIKEEIQRIKAGNPDISH

REAFSAAAKNWAHFPHIHFGLMADHPPTKKANVRQQEGEDGMMGREGFYGSAANVGVAHN

>GrYABBY1

MSTCTNLAPEQLCYIPCNFCNIVLAVSVPCSSLFDIVTVRCGHCTNLWSVNMAAAFQSLS

WQDVQVGASYATQDYRTDLGSSSKCNTKFSMRAPSKNATEERVVNRPPEKRQRVPSAYNQ

FIKEEIQRIKANNPDISHREAFSTAAKNWAHFPHIHFGLMLENQKSN

>GrYABBY2

MSMDLTSERVWYVHCNFCNTILAVSVPCNSAFNIVTVRCGHCANLLSVNMGTSLQTVPSQ

DAQKQQITIDEDHSNKECGSSSKCNKFSAVDSTEQEATRMPPIRPPEKRQRVPSAYNRFI

KEEIQRIKASNPDISHREAFSTAAKNWAHFPHIHFGLKLDGSKQAKLDQGYADQGSQKSN

GYC

>GrYABBY3

MSSSSAAFSPDHVSPSDQLCYVHCNFCDTVLAVSVPCTSLFKTVTVRCGHCTNLLSVNML

GLLLPSTNQHHVGHSFFTPHNLLEEIRSSATTNMVINQPNPNNSLMPPIHGGVEDIPKPL

VVNRPPEKRQRVPSAYNRFIKDEIQRIKAGNPDISHREAFSAAAKNWAHFPHIHFGLMPD

QPVKKTNVHQQEGEEMVMKDGFFAPNNVGVTPYQTSPNYC

>GrYABBY4

MSSSHNSAPEQLCYIPCNLCNIILAVNVPCNSLFETVTVRCGQCSNLCSVNLAASFQSRA

GKDVQVPNYTSSEYRIDLGSSSRCKNKLPKRATTINTTTQERVVNRPPDKRHRAPSLYNQ

FIKEEIQRIKLNNPDITHREAFSTAAKNWARFPHIHFGLMLETDNQPKLNDDSTEPFHQL

LK

>GrYABBY5

MSTLNHLFDLPEQICYVQCGFCTTILLVSVPCSSLSMVVTVRCGHCTSLLSVNMMKASFV

PLHLLASFAHDDEPKEGAVSEEVGGHRKTSDRRSPSLMSSSDNGEEDIVRVNPTVNKPPE

KRQRAPSAYNRFIKFIHSWWQRRDQETENSKSQHSAQRSLQHCCKKLGPLSSYSQAQQRR

RREL

>GrYABBY6

MNLEDKVGMDLVPQSEHLCYVRCNFCNTVLAVGIPCKRLLETVTVKCGHCSNLSFLSTRP

PLQGQCLDPQTSLTLQSFCGDFRKGTQFPSPSSSTSSEPSSPKAPFVVKPPEKKHRLPSA

YNRFMKEEIQRIKAANPEIPHREAFSAAAKNWARYIPNSPAASSVCGSSSNEQNDNV

>GrYABBY7

MSLDLASERVCYVHCNFCNTILAVSVPYNSLFKIVTVRCGHCANLLSVNMGTSQQIVPIQ

DAQKQQIINIDEDPSNKECGSSSKCNKQFSAFDSAQNEAPRMPPIRPPEKRQRVPSAYNR

FIKEEIQRIKASNPDISHREAFSTAAKNWAHFPHIHFGLKLEGNKQAKLDQSFADQGAQK

SNNYY

>GrYABBY8

MSSSSVSFSSDHLSPPDQLCYVHCNFCDTVLAVSVPCSSLFNNTVMVRCGHCTNLLSVDM

RGLLLPTANQLHLAHSFFTPQNLLEEIRSCAPPNMVMINHPNPNDTVIRGAAELEEIAKP

PVVNRPPEKRQRVPSAYNRFIKEEIQRIKAGNPDISHRDAFSAAAKNWAHFPRIHFGQMP

DQAVMKMKNNVRQQEGEEAVVKDGFFVPTNVGVSPY

>GrYABBY9

MSSSSTLSLDHLPPSEQLCYVHCNICDTVLAVSVPCTSLFKTVTVRCGHCTNLLPVNMRG

LLMPSANQFHLPHNFFTPSHNLLEEISNPSPNILLNQGNTSDITLPTRGVADELPRPPVI

NRPPEKRQRVPSAYNRFIKDEIQRIKAGNPDITHREAFSAAAKNWAHFPHIHFGLMPDQP

AKRTNVRQQEGEDVLMKDGFFASANVGVTPY

>GrYABBY10

MSNCIDVAPEQLCYIPCNFCNIVLAVSVPCSSLLDIVTVRCGHCTNLWSVNMAAAFQSVS

WQDIQAPNHAMQDYRMKESGSSSKCNKFPMRPPANNVVTEERVVNRPPGKRQRVPSAYNQ

FIKEEIQRIKANNPDISHREAFSTAAKNWAHFPHIHFGLMLEANNQTKLHDASEKHIMSR

TALFNK

>GrYABBY11

MSSLNNSAPEQLCYIPCNLCNIILAVNVPCSCLFETVTVRCGQCTNLCSINMAASFQSRA

GKEIQVPNYTSSEYRIELGSSSSKGKNKLPKRPRIMNTTTQERVVNRPPDKRHRAPSLYN

QFIKEEIQRIKLNNPDISHREAFSTAAKNWARFPHIHFGLMLETATQPKLNDDSTEHFQQ

LLK

>GrYABBY12

MSSCIEVVPEQLCYIPCNFCNIVLAVSVPCSSLFDIVTVRCGHCTNLWSVNMAAAASQSL

SWQDIQAPNNAIEDYRRDLGSSSKCSHNKLSMRPPLPNNATEERVVNRPPEKRQRVPSAY

NRFIKEEIQRIKANNPDISHREAFSTAAKNWAHFPHIHFGLMLETNNQTKMNNGSG

>GhYABBY4_At

MSSSHNSAPEQLCYIPCNLCNIILAVNVPCNSLFETVTVRCGQCTNLCSVNLAASFQSRA

GKDVQVPNYTSSEYRIDLGSSSRCKNKLPKRATTINTTTQERIVNRPPDKRHRAPSLYNQ

FIKEEIQRIKLNNPDITHREAFSTAAKNWARFPHIHFGLMLETDNQPKLNDDSTEPFHQL

LK

>GhYABBY5_At

MASDKEIKKVSVPCNSLSMVVTVRCGHCTSLLSVNMMKASFVPLHLLASFAHDDEPKEEA

VSEEVGGHRKTSDRRSPSLMSSSDNGEEDIVRVNPTVNKPPEKRQRAPSAYNRFIKEEIR

RLKTQNPNIPHKEAFSTAAKNWAHFPPIHKHNKGEGVSCERDEGEASWNVEATEVHIQGN

DFRERKAARNSIWTKSPFE

>GhYABBY6_At

MNLEDKVGMDLVPQSEHLCYVRCNFCNTVLAVGIPCKRLLETVTVKCGHCSNLSFLSTRP

PLQGQCLDPQTSLTLQSFCGDFRKGTQFPSPSSSTSSEPSSPKAPFVVKPPEKKHRLPSA

YNRFMKEEIQRIKAANPEIPHREAFSAAAKNWARYIPNSPAASSVCGSSSNVSNQIHYIY

IYNGKNVLIMGFGEGLL

>GhYABBY11_At

MSSFNNSAPEQLCYIPCNLCNIILAVNVPCSCLFETVTVRCGQCTNLCSINMAASFQSRA

GKEIQVPDYTSSEYRMELGSSSSKGKNKLPKRPTIMNTTTQERVVNRPPDKRHRAPSLYN

QFIKEEIQRIKLNNPDISHREAFSTAAKNWARFPHIHFGLMLETGTQPKLNDDSTEHFQQ

LLK

>GhYABBY10_At

MSNCIDVAPEQLCYIPCNFCNIVLAVSVPCSSLFDIVTVRCGHCTNLWSVNMAAAFQSLS

WQDIQAPNHAMQDYRMKESGSSSKCNKFPMRPPANNVVTEERVVNRPPGKRQRVPSAYNQ

FIKEEIQRIKANNPDISHREAFSTAAKNWAHFPHIHFGLMLEANNQTKLHDASEKHIMSR

TALFNK

>GhYABBY12_At

MSSCIEVVPEQLCYIPCNFCNIVLAVSVPCSSLFDIVTVRCGHCTNLWSVNMAAAASQSL

SWQDIQAPNNAIEDYRRDLGSSSKCSHNKLSMRPPLPNNATEERVVNRPPEKRQRVPSAY

NRFIKYDILNTINLYGRLNIQRIKANNPDISHREAFSTAAKNWAHFPHIHFGLMLETNNQ

TKMNNVSG

>GhYABBY1_At

MSTCTNVVPEQLCYIPCNFCNIVLAVSVPCSSLFDIVTVRCGHCTNLWSVNMAAAFQSLS

WQDVQAASYATQDYRTDLGSSSKCNTKFSMRAPSKNATEERVVNRPPEKRQRVPSAYNQF

IKEEIQRIKANNPDISHREAFSTAAKNWAHFPHIHFGLMLENQKSN

>GhYABBY2_At

MSMGLTSERVWYVHCNFCNTILAVSVPCNSAFNIVTVRCGHCANLLSVNMGTSLQTVPSQ

DAQKQQITIDEDHSNKECGSSSKCNKFSAVDSTEQEATRMPPIRPPEKRQRVPSAYNRFI

KEEIQRIKASNPDISHREAFSTAAKNWAHFPHIHFGLKLEGSKQAKLDQGYADQGSQKSN

GYC

>GhYABBY3_At

MSSSSAAFSPDHVSPSDQLCYVHCNFCDTVLAVSVPRTSLFKTVTVRCGHCTNLLSVNML

GLLLPTTNQHHVGHSFFTPHNLLEEIRSSATTNMVINQPNPNNSLMPPIHGGVEDIPKPP

VVNRPPEKRQRVPSAYNRFIKDEIQRIKAGNPDISHREAFSAAAKNWAHFPHIHFGLMPD

QPVKKTNVHQQEGEEMVMKDGFFAPNNVGVTPYQTSPNYC

>GhYABBY7_At

MSLDLASERVCYVHCNFCNTILAVSVPYNSLFKFVTVRCGHCANLLSVNMGTSQQTVPIQ

DAQKQQIINIDEDPSNKECGSSSKCNKQFSGFDSAQNEAPRMPPIRPPEKRQRVPSAYNR

FIKEEIQRIKASNPDISHREAFSTAAKNWAHFPHIHFGLKLEGNKQAKLDQSFADQGAQK

>GhYABBY8_At

MSSSSVSFSSDHLSPADQLCYVHCNFCDTVLAVSVPCSSLFNNTVMVRCGHCTNLLSVDM

RGLLLPTANQLHLAHSFFTPQNLLEEIRSCAPPNMVMINHPNPNDTVIRGAAELEEIAKP

PVVNRPPEKRQRVPSAYNRFIKEEIQRIKAGNPDISHRDAFSAAAKNWAHFPRIHFGQMP

DQAVMKMKNNVRQQEGEEAVVKDGFFVPTNVGVSPY

>GhYABBY9_At

MSSSSTLSLDHLPPSEQLCYVHCNICDTVLAVSVPCTSLFKTVTVRCGHCTNLLPVNMRG

LLMPSANQFHLPHNFFTPSHNLLEEISNPSPNILLNQGNTSDITLPTRGVVDELPRPPVI

NRPPEKRQRVPSAYNRFIKDEIQRIKAGNPDITHREAFSAAAKNWAHFPHIHFGLMPDQP

AKRTNVRQQESEDVLMKDGFFASANVGVTPY

>GhYABBY5_Dt

MSTLNHLFDLPEQICYVQCGFCTTILLVSVPRSSLSMVVTVRCGHCTSLLSVNMMKASFV

PLHLLASFAHDDEPKEGAVSEEVGGHRKTSDRRSPSLMSSSDNGEEDIVRVNPTVNKPPE

KRQRAPSAYNRFIKEEIRRLKTQNPNIPHKEAFSTAAKNWAHFPPIHKHNKGEGESCEQD

EGEASWNVEATEVGFKIICPFLGDAVGNEVFTASLY

>GhYABBY4_Dt

MSSSHNSAPEQLCYIPCNLCNIILAVNVPCNSLFETVTVRCGQCSNLCSVNLAASFQSRA

GKDVQVPNYTSSEYRIDLGSSSRCKNKLPKRATTINTTTQERVVNRPPDKRHRAPSLYNQ

FIKEEIQRIKLNNPDITHREAFSTAAKNWARFPHIHFGLMLETDNQPKLNDDSTEPFHQL

LK

>GhYABBY6_Dt

MNLEDKVGMDLVPQSEHLCYVRCNFCNTVLAVGIPCKRLLETVTVKCGHCSNLSFLSTRP

PLQGQCLDPQTSLTLQSFCGDFRKGTQFPSPSSSTSSEPSSPKAPFVVKPPEKKHRLPSA

YNRFMKEEIQRIKAANPEIPHREAFSAAAKNWARYIPNSPAASSVCGSSSNVSKQIHYIY

IMVKMF

>GhYABBY10_Dt

MSNCIDVAPEQLCYIPCNFCNIVLAVSVPCSSLFDIVTVRCGHCTNLWSVNMAAAFQSVS

WQDIQAPNHAMQDYRMKESGSSSKCNKFPMRPPANDVVTEERVVNRPPGKRQRVPSAYNQ

FIKEEIQRIKANNPDISHREAFSTAAKNWAHFPHIHFGLMLEANNQTKLHDASEKHIMSR

TALFNK

>GhYABBY11_Dt

MSSLNNSAPEQLCYIPCNLCNIILAVNVPCSCLFETVTVRCGQCTNLCSINMAASFQSRG

GKEIQVPNYTSSEYRIELGSSSSKGKNKLPKRPRIMNTTTQERVVNRPPDKRHRAPSLYN

QFIKEEIQRIKLNNPDISHREAFSTAAKNWARFPHIHFGLMLETATQPKLNDDSTEHFQQ

LLK

>GhYABBY12_Dt

MSSCIEVVSEQLCYIPCNFCNIVLAVSVPCSSLFDIVTVRCGHCTNLWSVNMAAAASQSL

SWQDIQAPNNAIEDYRRDLGSSSKCSHNKLSMRPPLPNNATEERVVNRPPEKRQRVPSAY

NRFIKYDILNTINLYGRLNIQRIKANNPDISHREAFSTAAKNWAHFPHIHFGLMLETNNQ

TKMNNVSG

>GhYABBY1_Dt

MSTCTNLAPEQLCYIPCNFCNIVLAVSVPCSSLFDSVTVRCGHCTNLWSVNMAAAFQSLS

WQDAQGASYATQDYRTDQGSSSKCNTKFSMRAPSKNATEERVVNRPPEKRQRVPSAYNQF

IKEEIQRIKANNPDISHREAFSTAAKNWAHFPHIHFGLMLENQKSN

>GhYABBY2_Dt

MSMDLTSERVWYVHCNFCNTILAVSVPCNSAFNIVTVRCGHCANLLSVNMGTSLQTVPSQ

DAQKQQITIDEDHSNKECGSSSKCNKFSAVDSTEQEATRMPPIRPPEKRQRVPSAYNRFI

KEEIQRIKASNPDISHREAFSTAAKNWAHFPHIHFGLKLDGSKQAKLDQGYADQGSQKSN

GYC

>GhYABBY3_Dt

MSSSSAAFSPDHVSPSDQLCYVHCNFCDTVLAVSVPCTSLFKTVTVRCGHCTNLLSVNML

GLLLPSTNQHHVGHSFFTPHNLLEEIRSSATTNMVINQPNPNNSLMPPIHGGVEDIPKPP

VVNRPPEKRQRVPSAYNRFIKDEIQRIKAGNPDISHREAFSAAAKNWAHFPLIHFGLMPD

QPVKKTNVHQQEGEEMVMKDGFFAPNNVGVTPYQTSPNYC

>GhYABBY8_Dt

MSSSSVSFSSDHLSPPDQLCYVHCNFCDTVLAVSVPCSSLFNNTVMVRCGHCTNLLSVDM

RGLLLPTANQLHLAHSFFTPQNLLEEIRSCGPPNMVMINHPNPNDTVIRGAAELEEIAKP

PVVNRPPEKRQRVPSAYNRFIKEEIQRIKAGNPDISHRDAFSAAAKNWAHFPRIHFGQMP

DQAVMKMKNNVRQQEGEEAVVKDGFFVPTNVGVSPY

>GhYABBY9_Dt

MSSSSTLSLDHLPPSEQLCYVHCNICDTVLAVSVPCTSLFKTVTVRCGHCTNLLPVNMRG

LLMPSANQFHLPHNFFTPSHNLLEEISNPSPNILLNQGNTSDITLPTRGVADELPRPPVI

NRPPEKRQRVPSAYNRFIKDEIQRIKAGNPDITHREAFSAAAKNWAHFPHIHFGLMPDQP

AKRTNVRQQEGEDVLMKDGFFASANVGVTPY

>GaYABBY7

MVSGDIRLGTLISSLRVSVPYNSLFKFVTVRCGHCANLLSVNMGTSQQTVPIQDAQKQQI

INIDEDPSNKECGSSSKCNKQFSGFDSAQNEAPRMPPIRPPEKRQRVPSAYNREEIQRIK

ASNPDISHREAFSTAAKNWAHFPHIHFGLKLEGNKQAKLDQSFADQGAQKSNPYY

>GaYABBY1

MRYKIRPAPPLRLSQGVSVPCSSLFDIVTVRCGHCTNLWSVNMAAAFQSLSWQDVQAASY

ATQDYRTDLGSSSKCNTKFSMRAPSKNATEERVVNRPPEKRQRVPSAYNQFIKEEIQRIK

ANNPDISHREAFSTAAKNWAHFPHIHFGLMLENQKSN

>GaYABBY9

MSSSSTLSLDHLPPSEQLCYVHCNICDTVLAVSVPCTSLFKTVTVRCGHCTNLLPVNMRG

LLMPSANQFHLPHNFFTPSHNLLEEISNPSPNILLNQGNTSDITLPTRGVVDELPRPPVI

NRPPEKRQRVPSAYNRFIKDEIQRIKAGNPDITHREAFSAAAKNWAHFPHIHFGLMPDQP

AKRTNESEDVLMKDGFFASANVGVTPY

>GaYABBY8

MSSSSVSFSSDHLSPADQLCYVHCNFCDTVLAVSVPCSSLFNNTVMVRCGHCTNLLSVDM

RGLLLPTANQLHLAHSFFTPQNLLEEIRSCAPPNMVMINHPNPNDTVIRGAAELEEIAKP

PVVNRPPEKRQRVPSAYNRFIKEEIQRIKAGNPDISHRDAFSAAAKNWAHFPRIHFGQMP

DQAVMKMKNNVRQQEGEEAVVKDGFFVPTNVGVSPY

>GaYABBY10

MSNCIDVAPEQLCYIPCNFCNIVLAVSVPCSSLFDIVTVRCGHCTNLWSVNMAAAFQSLS

WQDIQAPNHAMQDYRMKESGSSSKCNKFPMRPPANNVVTEERVVNRPPGKRQRVPSAYNQ

FIKEEIQRIKANNPDISHREAFSTAAKNWAHFPHIHFGLMLEANNQTKLHDASEKHIMSR

TALFNK

>GaYABBY12

MSSCIEVVPEQLCYIPCNFCNIVLAVSVPCSSLFDIVTVRCGHCTNLWSVNMAAAASQSL

SWQDIQAPNNAIEDYRRDLGSSSKCSHNKLSMRPPLPNNATEERVVNRPPEKRQRVPSAY

NRFIKEEIQRIKANNPDISHREAFSTAAKNVRKCMVVFENFDMKLWI

>GaYABBY2

MADLKTVSVPCNSAFNIVTVRCGHCANLLSVNMGTSLQTVPSQDAQKQQITIDEDHSNKE

CGSSSKCNKFSAVDSTEQEATRMPPIRPPEKRQRVPSAYNRFIKEEIQRIKASNPDISHR

EAFSTAAKNWAHFPHIHFGLKLEGSKQAKLDQGYADQGSQKSNGYC

>GaYABBY4

MSSSHNSAPEQLCYIPCNLCNIILAVFNSDPALVNVPCNSLFETVTVRCGQCTNLCSVNL

AASFQSRAGKDVQVPNYTSSEYRIDLGSSSRCKNKLPKRATTINTTTQERVVNRPPDKRH

RAPSLYNQFIKEEIQRIKLNNPDITHREAFSTAAKNWARFPHIHFGLMLETDNQPKLNDV

STNFHLEAMQTI

>GaYABBY3

MSSSSAAFSPDHVSPSDQLCYVHCNFCDTVLAVSVPCTSLFKTVTVRCGHCTNLLSVNML

GLLLPTTNQHHVGHSFFTPHNLLEEIRSSATTNMVINQPNPNNSLMPPIHGGAEDIPKPP

VVNRPPEKRQRVPSAYNRFIKDEIQRIKAGNPDISHREAFSAAAKNWAHFPHIHFGLMPD

QPVKKTNVHQQEGEEMVMKDGFFAPKNLMLVTMGMHFDVVKSLANMSSLSTKNSYEDEGF

DADEYLLLRH

>GaYABBY6

MNLEDKVGMDLVPQSEHLCYVRCNFCNTVLAVGIPCKRLLETVTVKCGHCSNLSFLSTRP

PLQGQCLDPQTSLTLQFVHVKEMQSFCGDFRKGTQFPSPSSSTSSEPSSPKAPFVVKPPE

KKHRLPSAYNRFMKEEIQRIKAANPEIPHREAFSAAAKNWARYIPNSPAASSVCGSSSNE

QNDNV

>Sobic.001G152901.1.p

NPFRHVQRGPNNSLWHFAPSTQAHKELKNREKPLRSLSLSLSLTHKHTQISSSISPLSFT

SLYLQAVCTTQGEKSRRNVGPADRAGAGACVLRALQLLQYNSRAPEKRQRVPSAYNRFIK

EEIRRIKASNPDISHREAFSTAAKNWAHFPNIHFGLGPYESSNKLDEAIGATGHPQKVQD

LY

>Sobic.001G199200.1.p

MSSSSSSSSAASAATVFPPSPQLPPSPLLVENLPPLHQLTPVAAAEAAAPASEQLCYVHC

HFCDTVLVVSVPTSSLFKTVTVRCGHCSSLLTVNMRGLLFPGTPTNTAAAAAPPAAVTAS

TTTTTTTTITTAPPPATSVNNGQFHFPQSVDLAPNPHHQSLLLDEISSANPRLQLLEQHG

LGGLIPSGRNAAAPAPPPPPAAAGKGAKEPSPRTNPVINRPPEKRQRVPSAYNRFIKDEI

QRIKAGNPDISHREAFSAAAKNWAHFPHIHFGLMPDHQGLKKTSLLPQDLQRKDGLLKEG

LYAAAAAAAAAAANMGVAPY

>Sobic.001G456000.1.p

MDMVSQSEHLCYVRCTYCNTVLALQVGVPCKRLMDTVTVKCGHCNNLSYLSPRPPMVQPL

SPTDHPLGPFQCQGPCNDCRRNQPLPLASPTSTELSPRMPFVVKPPEKKHRLPSAYNRFM

REEIQRIKAAKPDIPHREAFSMAAKNWAKCDPRCSTTASTATSNSAPEPRVVPTPQVTEA

RFDLEDRAKEQVIESFDIFKQIERSI

>Sobic.002G039400.1.p

MSAQFASEHACYVNCNYCNTILVVNVPNSCSHNIVTVKCGHCTMVLSMDLSPFHQQARTV

PDNQVVQNRGFQYNNFGSYEQASSRNLRTPSMYSVSNNQPQVPPIRPPEKRQRVPSAYNR

FIKEEIQRIKTSNPEISHREAFSAAAKNWAHLPRLHFGLSVADGGGGSN

>Sobic.002G346800.1.p

MSSSPRHPCFGALPERLGYVQCNLCATILLVGVPCGGTLQLLKTVAVQCGSCCGILSVAL

PPPAPASVELPLQEAGVGPPPRDSDESSGEDRETEATVADNHAAFPAVNKPPVRKQRTPS

AYNCFIKEEIQRIKARHPSITHKEAFSAASKNWAHLPRIQKKGE

>Sobic.004G302700.1.p

MMSSSSSSSAAFPLDHLAPSPTEQLCYVHCNCCDTILAVGVPCSSLFKTVTVRCGHCANL

LSVNLRGLLLPPAAPPANHLNFGHSLLSPTSPHGLLDELALQAPSLLMEQASANLSSSTM

TGGSNSSCASNLPAGPMPAAKPVQQEPELPKTTAPSVNRPPEKRQRVPSAYNRFIKDEIQ

RIKAGNPDITHREAFSAAAKNWAHFPHIHFGLMPDQGLKKTFKTQDGAEDMLLKDGLYAA

AAAAAAANMGITPF

>Sobic.006G160800.1.p

MMSSVPETFNLDQQHLVVQQQQPPPAEQEQICYVHCSYCDTILAVGVPCSSLFQTVTVRC

GHCSNLLYVNLRALLLPAAANQLPPFGGQALLSPTSPHGLLDAETMSSFQAPRSLQPSTD

PPSACVSTITSINNTCGGGNSASAMSSMAPPPPAKPALLEPQLPKSAASGNKTSEKRQRV

PSAYNRFIKDEIQRIKASNPDITHREAFSAAAKNWAHFPHIHFGLMPDQGLKKNPMQNQE

GAECMLFKDGLYAAAAAAAAAAAAATAASGMGISPF

>Sobic.008G176300.1.p

MSSAQIAPADHVCYVHCNFCNTVLAVSVPGNSMLNIVTVRCGHCTNLLSVNLRALMHSLP

EQDQLQENIKVHGVNGTLHDQCGHLELGSSSSSKFRLPMMYSPQNEHLLQEQTLNNARPP

EKRQRVPSAYNRFIKEEIRRIKANNPDISHREAFSTAAKNWAHYPNIHFGLNSGREGGKK

LVDEAVAAAPAPKKIQGFY

>Thecc1EG005549t1

MSSSSAAFSPDHLSPSDQLCYVHCNFCDTVLAVSVPCTSLFKTVTVRCGHCTNLLSVNMR

GLLLPAANQLHLGHSFFTPQNLLEEIRSAPPNMLINQPNPSDTVMPAIRGGVEEIPKPPV

VNRPPEKRQRVPSAYNRFIKDEIQRIKAGNPDISHREAFSAAAKNWAHFPHIHFGLMPDQ

PVKKTNVRQQEGEDVLMKDGFFASTNVGVTPY

>Thecc1EG010411t1

MSTLNHLFDLPEQICYVQCGFCTTILLVSVPCSSLSMVVTVRCGHCSSLLSVNMMKASFV

PLQLLASLGHDHDDEPKEGAVCEEVAAPRKTAAHRRSPSLMTSSDNEEEDIVRVNPTVNK

PPEKRQRAPSAYNRFIKLIPGGREEIRRLKAQNPNIPHKEAFSTAAKNWAHFPPMDTKGD

GESCGLDEGQALWNSDATQVLHVSRFDGKNKFTYKAMLSVKEKPQGVPYGPRHRLREDAL

DIDFY

>Thecc1EG011484t1

MPPKGPGSLVFFVHQEDNSVLEKDIKNPNLFSLLLKPTAPLFTSLAALCSILFSSLLFPS

LPFPSFDNMNLEEKVGMDLVPQSEHLCYVRCNFCNTVLAVGIPCKRLLDTVTVKCGHCSN

LSFLSTRPPLQGQCLDPQTSLSLQSFCGDFRKGQSPSPSSSPSSEPSSPKAPFVVKPPEK

KHRLPSAYNRFMKEEIQRIKAANPEIPHREAFSAAAKNWARYIPNSPATSVSGSRSNE

>Thecc1EG016030t1

MSSSSTLSLDHLPPSEQLCYVHCNICDTVLAVSVPCTSLFKTVTVRCGHCTNLLPVNMRG

LLLPSANQLHLAHSFFSPSHNLLEEISNPSQNFLLNQTNTNDFALPTRGVADELPRPPVI

NRPPEKRQRVPSAYNRFIKDEIQRIKAGNPDITHREAFSAAAKNWAHFPHIHFGLMPDQT

PKKTNVRQQEGEDVLIKDGFFASANVGVSPY

>Thecc1EG031868t1

MSSSNNSAPEQLCYIPCNLCNIILAVNVPCSSLFETVTVRCGQCTNLCSVNLATSFPSRA

GKDIQVPSYTSSEYRIDLGSSSRCKNKLPKRATTVNTTPERVVNRPPDKRHRAPSLYNQF

IKEEIQRIKLNNPDITHREAFSTAAKNWARFPHIHFGLMLETDNQPKLNDDSTEHFQQLL

K

>Thecc1EG038105t1

MSSCIDVAPEQLCYIPCNFCNIVLAVSVPCSSLFDIVTVRCGHCTNLWSVNMAAAFQSLS

WQDVQAPNYATQDYRTELGSSSKCNKLSMRTPANNVTEERVVNRPPEKRPRVPSAYNQFI

KEEIQRIKANNPDISHREAFSTAAKNWAHFPHIHFGLMLETNNQNKLDDASEKHLMSRTA

LLNK

>Thecc1EG041175t1

MSLDLSSERVCYVHCNFCNTILAVSVPCTSLFNIVTVRCGHCANLLSVNMGTSLQTVPIQ

DAQKEQINTDQDHSNKECGSSSKCNKFSAFDSAEHEAPRMPPIRPPEKRQRVPSAYNRFI

KEEIQRIKASNPDISHREAFSTAAKNWAHFPHIHFGLKLDGNKQAKLDQAFAEGTQKSTG

FY

>Os02g42950.1|OsYABBY4

MSSSSSSSAVFPLDHLAAPSPTEQLCYVHCNCCDTILAVGVPCSSLFKTVTVRCGHCANL

LSVNLRGLLLPAPAPAPANQLHFGPSLLSPTSPHGLLDEVAFQTPSLLMEQAASASLSSI

TGRSSSSCASNAPAMQMPPAKPVQQEPELPKNAPASANRPPEKRQRVPSAYNRFIKDEIQ

RIKAGNPDISHREAFSAAAKNWAHFPHIHFGLMPDQGFKKTFKPQDGSEDILLKDSLYAA

AAAAAAAAANMGVTPF

>Os03g11600.1|OsDL

MDLVSPSEHLCYVRCTYCNTVLALQVGVPCKRLMDTVTVKCGHCNNLSFLSPRPPMVQPL

SPTDHPLGPFQGPCTDCRRNQPLPLVSPTSNEGSPRAPFVVKPPEKKHRLPSAYNRFMRE

EIQRIKAAKPDIPHREAFSMAAKNWAKCDPRCSSTVSTSNSNPEPRVVAAPIPHQERANE

QVVESFDIFKQMERSG

>Os03g44710.1|OsYABBY2

MSAQIVPAPEHVCYVHCNFCNTIFAVSVPSNSMLNIVTVRCGHCTSLLSVNLRGLVQALP

AEDHLQDNLKMHNMSFRENYSEYGSSSRYGRVPMMFSKNDTEHMLHVRPPEKRQRVPSAY

NRFIKEEIRRIKANNPDISHREAFSTAAKNWAHFPNIHFGLGSHESSKKLDEAIGAPSPQ

KVQRLY

>Os04g45330.1|OsYABBY3

MMSSAPETFSLDHLSQHQQQQPPPLAEQEQLCYVHCNFCDTILAVGVPCSSLFKTVTVRC

GHCANLLSVNLRGLLLPAAASTANQLPFGQALLSPTSPHGLLDEVPSFQAPASLMTEQAS

PNVSSITSSNSSCANNAPATSMASAANKATQREPQQPKNAPSANRTSEKRQRVPSAYNRF

IKDEIQRIKASNPDITHREAFSAAAKNWAHFPHIHFGLMPDQGLKKTGIQSQDGAGECML

FKDGLYAAAAAAAAATAASSMGVTPF

>Os07g06620.1|OsYABBY1

MSVQFTSEHVCYVNCNYCNTILVVNVPNNCSYNIVTVRCGHCTMVLSMDLAPFHQARTVQ

DHQVQNRGFQGNNFGSYDIASRNQRTSTAMYPMPTSQQQVSPIRPPEKRQRVPSAYNRFI

KEEIQRIKTSNPEISHREAFSAAAKNWAHLPRLHFGLSVADGGGGGGSN

>Os07g38410.1|OsYABBY7

MSSAARHHCSGLRERLGCVQCSFCATVLLVSVPCSSVLRVVAVQCGHCSGILSAVNLPPS

PVSASIELTPQELDAGPPPGEYSDESSGDDREGRDAEDDAPAPAAAAVANKPPGRKQRTP

SAYNCFVKEEIKRIKSMEPNITHKQAFSTAAKNWAHLPRIQQKRGRDSC

>Os10g36420.1|OsYABBY3

MSSSSSSSASSAAAAAFRPAVVQREQQVVEEKFPAAAAAMREMVLPPVAAAAADSEQEQL

CYVHCHYCDTVLVVSVPSSSLFETVTVRCGHCSSLLTVNMRGLLLPTTAAAAPPPPPPPP

PPPPPPAAHFPHSLNLAPANPPHHHSLLDEISTANSPTQLLLEQHGLGGLMASAASCRNN

NSPAAAAAPPPPTSQGKAAAKEPSPRTNTAVINRPPEKRQRVPSAYNRFIKDEIQRIKAG

NPDISHREAFSAAAKNWAHFPHIHFGLMPDHQGLKKTSLLPQDHQRKDGLLKEGLYAAAA

AAAAAANMGVAPY

>Os12g42610.1|OsYABBY6

MSAQIAPAEQVCYVHCNFCNTILAVSVPGNSMLNIVTVRCGHCTNLLSVNLRGLMHSAPA

LQDHHHHHLQESGLSGCFRDQSGYPEFGFSAASSSSKLRLPPAAAAMVSYSQQNQQLEQA

LHARPPEKRQRVPSAYNRFIKEEIRRIKANNPDISHREAFSTAAKNWAHYPNIHFGLSPG

HEGGKKLVDVDPIPTAPSSKKIQGFYS

>GRMZM2G005353_P01

MTSPVPETFSLQDQQPPPPPPAEQEQICYVHCSYCDTILAVGVPCSSLFQTVTVRCGHCA

NLLYVNLRALLLPPATAPAAANHLPPFGQALLSPTSPHGLLDAETMSSSSFQAPSLPSAE

PPSAACVSGITSINNTACGGNNAASAMAPPPAKPALHEPPQLPRSAASANKTSEKRQRVP

SAYNRFIKDEIQRIKASNPDITHREAFSAAAKNWAHFPHIHFGLMPDQGLKKHPMQTQEG

AECMLFKDGLYAAAAAATAASSMGISPF

>GRMZM2G046829_P01

MSSSSSPRHPCFGAALPERLGYVQCKFCATILLVGVPIGGSLQLLKTVAVQCGSCCGILS

VALPPDEAPAPASVELLPLMQEAGGVDPPPRDSDESSGEDRGETTEAATVADNHAAFPAV

NKPPLRKQRTPSAYNCFIKEEIQRIKARDPGITHKEAFSAASKNWAHLPRIQKTGG

>GRMZM2G054795_P01

MMSSAPEETLFNLDQQQPPPAEQEQICYVHCSYCDTILAVGVPCSSLFQTVTVRCGHCSN

LLYVNLRALLLPAAAANNQLPPFGQPLLSPTSPHGLLDAEAMSFQAPSLPGAEPPSACVS

GVTSINNTCGGNTTTSSAMSSMAPPPAKHALQEAQQLPRTAASVNRTSEKRQRVPSAYNR

FIKDEIQRIKASNPDITHREAFSAAAKNWAHFPHIHFGLMPDQGLKKNPMQNQEGAECML

FMDGLYASMGFSPF

>GRMZM2G074543_P01

MHRSDVSLLLFFFLLALSLGEYDCRDEIQRIKAGNPNISHREAFSAAAKNWAHFPHIHFG

LMPDHQGLKTTSLLPQDHQRKDGLLKEGLYAAAAAAAAHAAANMGIAPY

>GRMZM2G085873_P01

MLNMVTVRCGHCTSLLSVNLRGLIQSLPVVQNHYSQEHFKVQNFSFTENYPEYAAPPSSS

RYRMPTMLSAKGDLDHMLHVRAPEKRQRVPSAYNRFIKEEIRRIKANNPDISHREAFSTA

AKNWAHFPNIHFGLGPYESSNKLDETIGATGHPRKIQDPY

>GRMZM2G088309_P01

MDMVSQSEHLCYVRCTYCNTVLAVGVPCKRLMDTVTVKCGHCNNLSYLSPRPPMVQPLSP

TDHPLGPFQCQGPCNDCRRNQPLPLASPSSTELSPRMPFVVKPPEKKHRLPSAYNRFMRE

EIQRIKAAKPDIPHREAFSMAAKNWAKCDPRCSTAASTETSNSAPAEPRVVPTPQLTEPR

FDLEDRAKGQVIESFDIFKHIERSI

>GRMZM2G102218_P01

MCAWGPGIIAGIETLPPSPLVACSTDSSSSSSTSRPVSSPFFVCLCLLVSRLTTQLASRQ

PDTRCFQISHRTVVVGPSENIPPRPPPRAPPRPRTPAGHQASMDTVSQSEHLCYVRCTYC

NTVLAVGVPCKRLMDTVTVKCGHCNNLSYLSPRPPMVQPLSPTDHPLGPFQCQGPCSECR

RNQPLPLASPTSTDLTPRMPFVVKPPEKKHRLPSAYNRFMREEIQRIKAAKPDIPHREAF

SMAAKNWAKCDPRCSTTASTATSNSAPEPARVVVPTPHVTEPRFDLEDRAKEHVIESFDI

FKQIERNI

>GRMZM2G106204_P01

MSAQFASEHACYVNCNYCNTILVVNVPSSCSYNVVTVKCGHCTMVLSMDLSPFHQQARTV

PDNQVVQNRGFQYNNFGSYEQASSRNLRTPPMYPVSNNQPQVPPIRPSEKRQRVPSAYNR

FIKEEIQRIKTSNPEISHREAFSAAAKNWAHLPRLHFGLSVADGGGGSN

>GRMZM2G116646_P01

MLNIVTVRCGHCANLLSVNLRALMHSLPEQDHQLQQENIKVHGINGTLHDDHQYCGHLDQ

LGSSSSSRFRRLPVMCSPQNEQHLLQEQTLNNNARPPEKRQRVPSAYNRFIKEEIRRIKA

NNPDINHREAFSTAAKNWAHYPNIHFGLDDSGREGKKKLVDHEAAASVVAVAAAPTAAKK

IQGFY

>GRMZM2G141955_P01

MSSAQIAPADHVCYVHCNFCNTVLAVSVPGNSMLSMVTVRCGHCTNLLSVNLRALMHSVP

EQDQLQENIRVHGTLREHHQCGGGHHLELGSSSSSRFRLPMMMSYAPQNEHLLQEQTLNN

ARPAPEKRQRVPSAYNRFIKEEIRRIKANNPDISHREAFSTAAKNWAHYPNIHFGLNSGR

EGGKNKLVDEAVAAVAVAPKKIQGFY

>GRMZM2G167824_P01

MSSSSSSSSAATVFPPSPQLPPPLLVENLPPLHQLTPPVAAAAAPASEQLCYVHCHFCDT

VLVVSVPTSSLFKTVTVRCGHCSSLLTVNMRGLLFPGTPANTAAAAAAAPPPPPAAAVTS

TTATMTTAPPPPPATSVNNNGQFHFIPHSLDLALPIPPHQSLLLDEISSAANPSLQLLEQ

HGLGGMITSGRNAAAPHPHPPQPQAPAAGKGAKEPSPRANSAINRPPEKRQRVPSAYNRF

IKDEIQRIKAGNPDISHREAFSAAAKNWAHFPHIHFGLMPDHQGPKKTSLLPQDHQRSDG

GGLLKEGLYAAAANMGVAPY

>GaYABBY11

MSSFNNSAPEQLCYIPCNLCNIILAVNVPCSCLFETVTVRCGQCTNLCSINMAASFQSRAGKEIQVPNYTSSEYRIELGSSSSKGKNKLPKRPTIMNTTTQERVVNRPPDKRHRAPSLYNQFIKEEIQRIKLNNPDISHREAFSTAAKNWARFPHIHFGLMLETGTQPKLNDDSTEHFQQLLK

>GaYABBY5

MSTLNHLFDLPEQICYVQCGFCTTILLVSVPCNSLSMVVTVRCGHCTSLLSVNMMKASFVPLHLLASFAHDDEPKEEAVSEEVGGHRKTSDRRSPSLMSSSDNGEEDIVRVNPTVNKPPEKRQRAPSAYNRFIKEEIRRLKTQNPNIPHKEAFSTAAKNWAHFPPIHKHNKGEGVSCERDEGEASWNVEATEVHIQGNDFRERKAARNSIWTKSPFE
