# Supplementary material for: Genome-Wide Study of YABBY Genes in Upland Cotton and Their Expression Patterns under Different Stresses
Source: Front Genet. 2018 Feb 7;9:33. doi: 10.3389/fgene.2018.00033 (PMC5808293; doi:10.3389/fgene.2018.00033)
Supplement: Supplementary file 2 [file Table2.DOCX]

| Gene name | Gene locus | Chr | Direction | Start | End |
| --- | --- | --- | --- | --- | --- |
| GrYABBY1 | Gorai.001G042700.1 | Chr01 | - | 4030104 | 4033712 |
| GrYABBY2 | Gorai.001G127000.1 | Chr01 | + | 16010195 | 16016122 |
| GrYABBY3 | Gorai.001G174800.1 | Chr01 | + | 25604586 | 25607592 |
| GrYABBY4 | Gorai.002G054200.1 | Chr02 | + | 4802568 | 4807218 |
| GrYABBY5 | Gorai.002G185900.1 | Chr02 | - | 49442964 | 49444942 |
| GrYABBY6 | Gorai.005G146700.1 | Chr05 | + | 40018671 | 40020740 |
| GrYABBY7 | Gorai.006G117800.1 | Chr06 | + | 36726690 | 36732764 |
| GrYABBY8 | Gorai.007G089700.1 | Chr07 | - | 6528845 | 6531148 |
| GrYABBY9 | Gorai.008G236500.1 | Chr08 | + | 52221505 | 52223852 |
| GrYABBY10 | Gorai.009G016700.1 | Chr09 | + | 1318103 | 1321175 |
| GrYABBY11 | Gorai.009G367100.1 | Chr09 | + | 49185118 | 49189637 |
| GrYABBY12 | Gorai.010G062900.1 | Chr10 | + | 7843388 | 7846876 |
| GaYABBY1 | Cotton_A_03518 | Ca1 | - | 65896175 | 65899452 |
| GaYABBY2 | Cotton_A_20510 | Ca1 | + | 58517967 | 58521699 |
| GaYABBY3 | Cotton_A_35599 | Ca1 | + | 143054542 | 143057066 |
| GaYABBY4 | Cotton_A_29420 | Ca4 | - | 93000332 | 93003458 |
| GaYABBY5 | Cotton_A_40397 | scaffold3840 | + | 31658 | 33443 |
| GaYABBY6 | Cotton_A_38604 | Ca1 | - | 126977084 | 126978694 |
| GaYABBY7 | Cotton_A_01709 | Ca10 | + | 78056940 | 78060962 |
| GaYABBY8 | Cotton_A_07719 | Ca2 | + | 114624338 | 114625991 |
| GaYABBY9 | Cotton_A_07122 | Ca9 | + | 74434202 | 74436302 |
| GaYABBY10 | Cotton_A_11462 | Ca6 | - | 69358715 | 69361140 |
| GaYABBY11 | Cotton_A_37397 | scaffold1680 |  | 235264 | 239525 |
| GaYABBY12 | Cotton_A_17419 | Ca11 | - | 28024301 | 28026477 |
| GhYABBY1_Dt | Gh_D07G0365 | D07 | - | 3914516 | 3917055 |
| GhYABBY2_Dt | Gh_D07G1125 | D07 | + | 16490252 | 16495415 |
| GhYABBY3_Dt | Gh_D07G1471 | D07 | + | 25363684 | 25365957 |
| GhYABBY4_Dt | Gh_D01G2326 | scaffold3715_D01 | - | 62778 | 66234 |
| GhYABBY5_Dt | Gh_D01G1535 | D01 | - | 47403009 | 47404981 |
| GhYABBY6_Dt | Gh_D02G1305 | D02 | + | 42994506 | 42996043 |
| GhYABBY8_Dt | Gh_D11G0842 | D11 | - | 7279031 | 7280682 |
| GhYABBY9_Dt | Gh_D12G2170 | D12 | + | 54762452 | 54764557 |
| GhYABBY10_Dt | Gh_D05G0144 | D05 | + | 1462286 | 1464684 |
| GhYABBY11_Dt | Gh_D05G3293 | D05 | + | 52515750 | 52518786 |
| GhYABBY12_Dt | Gh_D06G0514 | D06 | + | 7776266 | 7778251 |
| GhYABBY1_At | Gh_A07G0308 | A07 | - | 3818249 | 3820644 |
| GhYABBY2_At | Gh_A07G1044 | A07 | + | 20600022 | 20605191 |
| GhYABBY3_At | Gh_A07G1363 | A07 | + | 34508268 | 34510559 |
| GhYABBY4_At | Gh_A01G0376 | A01 | + | 5553159 | 5556688 |
| GhYABBY5_At | Gh_A01G1348 | A01 | - | 83639857 | 83637887 |
| GhYABBY6_At | Gh_A03G0924 | A03 | + | 58745520 | 58747078 |
| GhYABBY7_At | Gh_A09G0958 | A09 | + | 59175266 | 59180021 |
| GhYABBY8_At | Gh_A11G0723 | A11 | - | 7019946 | 7021599 |
| GhYABBY9_At | Gh_A12G1991 | A12 | + | 82721749 | 82723862 |
| GhYABBY10_At | Gh_A05G0082 | A05 | + | 979987 | 982406 |
| GhYABBY11_At | Gh_A04G0351 | A04 | - | 9063895 | 9068077 |
| GhYABBY12_At | Gh_A06G0472 | A06 | + | 8985093 | 8987085 |

**Supplementary Table 2. Gene loci information pertaining to cotton YABBY genes**
