# Supplementary material for: Genome-Wide Study of YABBY Genes in Upland Cotton and Their Expression Patterns under Different Stresses
Source: Front Genet. 2018 Feb 7;9:33. doi: 10.3389/fgene.2018.00033 (PMC5808293; doi:10.3389/fgene.2018.00033)
Supplement: Supplementary file 3 [file Table3.DOCX]

**Supplementary Table 3. Tblastn and blastn results for *GhYABBY7_Dt***

# tblastn result

# using the protein sequence of GhYABBY7_At to search the genomic sequene

GhYABBY7_At D09 84.44 45 7 0 115 159 35588685 35588819 6e-16 81.6

GhYABBY7_At A07 82.50 40 7 0 119 158 20605066 20605185 6e-14 75.9

GhYABBY7_At A07 83.33 42 7 0 22 63 20601637 20601762 3e-12 70.5

GhYABBY7_At D07 80.00 40 8 0 119 158 16495290 16495409 2e-13 74.3

GhYABBY7_At D07 83.33 42 7 0 22 63 16491879 16492004 4e-12 70.5

GhYABBY7_At scaffold73742 94.12 34 2 0 21 54 523 624 2e-12 67.8

# blastn result

#using the genomic sequence of GhYABBY7 to search the G. hirsutum genomic database

GhYABBY7_At D09 97.30 111 3 0 367 477 35588709 35588819 1e-45 189

GhYABBY7_At scaffold73742 97.14 105 1 2 60 163 522 625 8e-42 176

GhYABBY7_At D07 90.91 121 11 0 69 189 16491884 16492004 6e-38 163

GhYABBY7_At D07 90.10 101 10 0 367 467 16495302 16495402 2e-28 132

GhYABBY7_At D07 84.85 132 14 2 188 319 16492431 16492556 2e-27 128

GhYABBY7_At D07 94.37 71 4 0 1 71 16490252 16490322 8e-22 110

GhYABBY7_At A07 90.08 121 12 0 69 189 20601642 20601762 3e-36 158

GhYABBY7_At A07 92.08 101 8 0 367 467 20605078 20605178 8e-32 143

GhYABBY7_At A07 92.96 71 5 0 1 71 20600022 20600092 4e-20 104

GhYABBY7_At scaffold216407 100.00 71 0 0 1 71 8 78 2e-28 132

GhYABBY7_At scaffold55171 84.85 132 14 2 188 319 487 612 2e-27 128

GhYABBY7_At scaffold55171 95.00 60 3 0 130 189 1 60 2e-17 95.3

GhYABBY7_At scaffold200624 100.00 47 0 0 320 366 248 202 4e-15 87.9
