# Supplementary material for: Genome-Wide Study of YABBY Genes in Upland Cotton and Their Expression Patterns under Different Stresses
Source: Front Genet. 2018 Feb 7;9:33. doi: 10.3389/fgene.2018.00033 (PMC5808293; doi:10.3389/fgene.2018.00033)
Supplement: Supplementary file 4 [file Table4.DOCX]

**Supplementary Table 4. The *GhYABBY11_At* isoform prediction using AUGUSTUS**

# This output was generated with AUGUSTUS (version 3.3).

# AUGUSTUS is a gene prediction tool written by M. Stanke (mario.stanke@uni-greifswald.de),

# O. Keller, S. KÃ¶nig, L. Gerischer and L. Romoth.

# Please cite: Mario Stanke, Mark Diekhans, Robert Baertsch, David Haussler (2008),

# Using native and syntenically mapped cDNA alignments to improve de novo gene finding

# Bioinformatics 24: 637-644, doi 10.1093/bioinformatics/btn013

# No extrinsic information on sequences given.

# Initialising the parameters using config directory /data/www/augustus/augustus/config/ ...

# tomato version. Using default transition matrix.

# Looks like /data/www/augustus/tmp/AUG-1965460624/input.fa is in fasta format.

# We have hints for 0 sequences and for 0 of the sequences in the input set.

#

# ----- prediction on sequence number 1 (length = 3545, name = D05_52515750_52519294) -----

#

# Constraints/Hints:

# (none)

# Predicted genes for sequence number 1 on both strands

# start gene g1

D05_52515750_52519294 AUGUSTUS gene 1 3545 0.25 + . g1

D05_52515750_52519294 AUGUSTUS transcript 1 3545 0.21 + . g1.t1

D05_52515750_52519294 AUGUSTUS start_codon 1 3 . + 0 transcript_id "g1.t1"; gene_id "g1";

D05_52515750_52519294 AUGUSTUS initial 1 75 0.98 + 0 transcript_id "g1.t1"; gene_id "g1";

D05_52515750_52519294 AUGUSTUS internal 930 1049 0.91 + 0 transcript_id "g1.t1"; gene_id "g1";

D05_52515750_52519294 AUGUSTUS internal 2302 2428 1 + 0 transcript_id "g1.t1"; gene_id "g1";

D05_52515750_52519294 AUGUSTUS internal 2516 2564 0.89 + 2 transcript_id "g1.t1"; gene_id "g1";

D05_52515750_52519294 AUGUSTUS internal 2669 2744 0.89 + 1 transcript_id "g1.t1"; gene_id "g1";

D05_52515750_52519294 AUGUSTUS internal 2885 2953 0.62 + 0 transcript_id "g1.t1"; gene_id "g1";

D05_52515750_52519294 AUGUSTUS terminal 3510 3545 0.36 + 0 transcript_id "g1.t1"; gene_id "g1";

D05_52515750_52519294 AUGUSTUS intron 76 929 0.88 + . transcript_id "g1.t1"; gene_id "g1";

D05_52515750_52519294 AUGUSTUS intron 1050 2301 0.93 + . transcript_id "g1.t1"; gene_id "g1";

D05_52515750_52519294 AUGUSTUS intron 2429 2515 1 + . transcript_id "g1.t1"; gene_id "g1";

D05_52515750_52519294 AUGUSTUS intron 2565 2668 0.89 + . transcript_id "g1.t1"; gene_id "g1";

D05_52515750_52519294 AUGUSTUS intron 2745 2884 0.67 + . transcript_id "g1.t1"; gene_id "g1";

D05_52515750_52519294 AUGUSTUS intron 2954 3509 0.23 + . transcript_id "g1.t1"; gene_id "g1";

D05_52515750_52519294 AUGUSTUS CDS 1 75 0.98 + 0 transcript_id "g1.t1"; gene_id "g1";

D05_52515750_52519294 AUGUSTUS exon 1 75 . + . transcript_id "g1.t1"; gene_id "g1";

D05_52515750_52519294 AUGUSTUS CDS 930 1049 0.91 + 0 transcript_id "g1.t1"; gene_id "g1";

D05_52515750_52519294 AUGUSTUS exon 930 1049 . + . transcript_id "g1.t1"; gene_id "g1";

D05_52515750_52519294 AUGUSTUS CDS 2302 2428 1 + 0 transcript_id "g1.t1"; gene_id "g1";

D05_52515750_52519294 AUGUSTUS exon 2302 2428 . + . transcript_id "g1.t1"; gene_id "g1";

D05_52515750_52519294 AUGUSTUS CDS 2516 2564 0.89 + 2 transcript_id "g1.t1"; gene_id "g1";

D05_52515750_52519294 AUGUSTUS exon 2516 2564 . + . transcript_id "g1.t1"; gene_id "g1";

D05_52515750_52519294 AUGUSTUS CDS 2669 2744 0.89 + 1 transcript_id "g1.t1"; gene_id "g1";

D05_52515750_52519294 AUGUSTUS exon 2669 2744 . + . transcript_id "g1.t1"; gene_id "g1";

D05_52515750_52519294 AUGUSTUS CDS 2885 2953 0.62 + 0 transcript_id "g1.t1"; gene_id "g1";

D05_52515750_52519294 AUGUSTUS exon 2885 2953 . + . transcript_id "g1.t1"; gene_id "g1";

D05_52515750_52519294 AUGUSTUS CDS 3510 3545 0.36 + 0 transcript_id "g1.t1"; gene_id "g1";

D05_52515750_52519294 AUGUSTUS exon 3510 3545 . + . transcript_id "g1.t1"; gene_id "g1";

D05_52515750_52519294 AUGUSTUS stop_codon 3543 3545 . + 0 transcript_id "g1.t1"; gene_id "g1";

# coding sequence = [atgtcaagcctcaacaattctgcaccagaacaactgtgctacatcccttgcaacctttgcaacatcattcttgcggtga

# atgttccctgcagctgcttgtttgaaactgtgacagttcgatgtgggcagtgcaccaatctgtgttcaataaacatggcagcttcctttcaatccaga

# ggtggaaaagagatccaggtgcctaactacacatcatcggagtatagaattgagttagggtcttcttcttccaaaggcaagaacaagttaccaaagcg

# accaagaattatgaatactaccactcaagaaagggttgtgaaccgacctcctgacaagaggcatagggcaccttcattgtacaatcagttcatcaaag

# aggagattcaaaggatcaagctgaacaatcctgatatcagtcatagggaggcatttagcactgctgccaaaaattgggcacgcttccctcacattcat

# tttggactgatgttggagactgctactcaacctaagctgaatgatgactcgactgagcatttccagcagctccttaagtga]

# protein sequence = [MSSLNNSAPEQLCYIPCNLCNIILAVNVPCSCLFETVTVRCGQCTNLCSINMAASFQSRGGKEIQVPNYTSSEYRIEL

# GSSSSKGKNKLPKRPRIMNTTTQERVVNRPPDKRHRAPSLYNQFIKEEIQRIKLNNPDISHREAFSTAAKNWARFPHIHFGLMLETATQPKLNDDSTE

# HFQQLLK]

D05_52515750_52519294 AUGUSTUS transcript 1 3110 0.04 + . g1.t2

D05_52515750_52519294 AUGUSTUS start_codon 1 3 . + 0 transcript_id "g1.t2"; gene_id "g1";

D05_52515750_52519294 AUGUSTUS initial 1 75 0.98 + 0 transcript_id "g1.t2"; gene_id "g1";

D05_52515750_52519294 AUGUSTUS internal 930 1049 0.91 + 0 transcript_id "g1.t2"; gene_id "g1";

D05_52515750_52519294 AUGUSTUS internal 2302 2428 1 + 0 transcript_id "g1.t2"; gene_id "g1";

D05_52515750_52519294 AUGUSTUS internal 2516 2564 0.89 + 2 transcript_id "g1.t2"; gene_id "g1";

D05_52515750_52519294 AUGUSTUS internal 2669 2744 0.89 + 1 transcript_id "g1.t2"; gene_id "g1";

D05_52515750_52519294 AUGUSTUS internal 2885 2953 0.62 + 0 transcript_id "g1.t2"; gene_id "g1";

D05_52515750_52519294 AUGUSTUS terminal 3031 3087 0.26 + 0 transcript_id "g1.t2"; gene_id "g1";

D05_52515750_52519294 AUGUSTUS intron 76 929 0.88 + . transcript_id "g1.t2"; gene_id "g1";

D05_52515750_52519294 AUGUSTUS intron 1050 2301 0.93 + . transcript_id "g1.t2"; gene_id "g1";

D05_52515750_52519294 AUGUSTUS intron 2429 2515 1 + . transcript_id "g1.t2"; gene_id "g1";

D05_52515750_52519294 AUGUSTUS intron 2565 2668 0.89 + . transcript_id "g1.t2"; gene_id "g1";

D05_52515750_52519294 AUGUSTUS intron 2745 2884 0.67 + . transcript_id "g1.t2"; gene_id "g1";

D05_52515750_52519294 AUGUSTUS intron 2954 3030 0.21 + . transcript_id "g1.t2"; gene_id "g1";

D05_52515750_52519294 AUGUSTUS CDS 1 75 0.98 + 0 transcript_id "g1.t2"; gene_id "g1";

D05_52515750_52519294 AUGUSTUS exon 1 75 . + . transcript_id "g1.t2"; gene_id "g1";

D05_52515750_52519294 AUGUSTUS CDS 930 1049 0.91 + 0 transcript_id "g1.t2"; gene_id "g1";

D05_52515750_52519294 AUGUSTUS exon 930 1049 . + . transcript_id "g1.t2"; gene_id "g1";

D05_52515750_52519294 AUGUSTUS CDS 2302 2428 1 + 0 transcript_id "g1.t2"; gene_id "g1";

D05_52515750_52519294 AUGUSTUS exon 2302 2428 . + . transcript_id "g1.t2"; gene_id "g1";

D05_52515750_52519294 AUGUSTUS CDS 2516 2564 0.89 + 2 transcript_id "g1.t2"; gene_id "g1";

D05_52515750_52519294 AUGUSTUS exon 2516 2564 . + . transcript_id "g1.t2"; gene_id "g1";

D05_52515750_52519294 AUGUSTUS CDS 2669 2744 0.89 + 1 transcript_id "g1.t2"; gene_id "g1";

D05_52515750_52519294 AUGUSTUS exon 2669 2744 . + . transcript_id "g1.t2"; gene_id "g1";

D05_52515750_52519294 AUGUSTUS CDS 2885 2953 0.62 + 0 transcript_id "g1.t2"; gene_id "g1";

D05_52515750_52519294 AUGUSTUS exon 2885 2953 . + . transcript_id "g1.t2"; gene_id "g1";

D05_52515750_52519294 AUGUSTUS CDS 3031 3087 0.26 + 0 transcript_id "g1.t2"; gene_id "g1";

D05_52515750_52519294 AUGUSTUS exon 3031 3110 . + . transcript_id "g1.t2"; gene_id "g1";

D05_52515750_52519294 AUGUSTUS stop_codon 3085 3087 . + 0 transcript_id "g1.t2"; gene_id "g1";

D05_52515750_52519294 AUGUSTUS tts 3110 3110 . + . transcript_id "g1.t2"; gene_id "g1";

# coding sequence = [atgtcaagcctcaacaattctgcaccagaacaactgtgctacatcccttgcaacctttgcaacatcattcttgcggtga

# atgttccctgcagctgcttgtttgaaactgtgacagttcgatgtgggcagtgcaccaatctgtgttcaataaacatggcagcttcctttcaatccaga

# ggtggaaaagagatccaggtgcctaactacacatcatcggagtatagaattgagttagggtcttcttcttccaaaggcaagaacaagttaccaaagcg

# accaagaattatgaatactaccactcaagaaagggttgtgaaccgacctcctgacaagaggcatagggcaccttcattgtacaatcagttcatcaaag

# aggagattcaaaggatcaagctgaacaatcctgatatcagtcatagggaggcatttagcactgctgccaaaaattgggcacgcttccctcacattcat

# tttggactgatgttggagactgctactcaacctaagctgaatgatgaattgatcttatatgttttggaaaaaggggagggaagggctcaattccatgc

# ctaa]

# protein sequence = [MSSLNNSAPEQLCYIPCNLCNIILAVNVPCSCLFETVTVRCGQCTNLCSINMAASFQSRGGKEIQVPNYTSSEYRIEL

# GSSSSKGKNKLPKRPRIMNTTTQERVVNRPPDKRHRAPSLYNQFIKEEIQRIKLNNPDISHREAFSTAAKNWARFPHIHFGLMLETATQPKLNDELIL

# YVLEKGEGRAQFHA]

# end gene g1

###

# command line:

# /data/www/augustus/augustus/bin/augustus --species=tomato --strand=both --singlestrand=false --genemodel=partial --codingseq=on --sample=100 --keep_viterbi=true --alternatives-from-sampling=true --minexonintronprob=0.2 --minmeanexonintronprob=0.5 --maxtracks=2 /data/www/augustus/tmp/AUG-1965460624/input.fa --exonnames=on

Done.

submit another job
