# Supplementary material for: Genome-Wide Study of YABBY Genes in Upland Cotton and Their Expression Patterns under Different Stresses
Source: Front Genet. 2018 Feb 7;9:33. doi: 10.3389/fgene.2018.00033 (PMC5808293; doi:10.3389/fgene.2018.00033)
Supplement: Supplementary file 5 [file Table5.DOCX]

**Supplementary Table 5. The transposable element information pertaining to duplicated genes**

| Gene name | Gene | Gene_type | Chromosome |
| --- | --- | --- | --- |
| GhYABBY1_At | Gh_A07G0308 | 4 | A07 |
| GhYABBY2_At | Gh_A07G1044 | 4 | A07 |
| GhYABBY3_At | Gh_A07G1363 | 0 | A07 |
| GhYABBY4_At | Gh_A01G0376 | 4 | A01 |
| GhYABBY5_At | Gh_A01G1348 | 0 | A01 |
| GhYABBY6_At | Gh_A03G0924 | 0 | A03 |
| GhYABBY7_At | Gh_A09G0958 | 4 | A09 |
| GhYABBY8_At | Gh_A11G0723 | 0 | A11 |
| GhYABBY9_At  GhYABBY10_At  GhYABBY11_At  GhYABBY12_At | Gh_A12G1991  Gh_A05G0082  Gh_A04G0351  Gh_A06G0472 | 0  4  4  4 | A12  A05  A04  A06 |
| GhYABBY1_Dt | Gh_D07G0365 | 4 | D07 |
| GhYABBY2_Dt | Gh_D07G1125 | 0 | D07 |
| GhYABBY3_Dt | Gh_D07G1471 | 0 | D07 |
| GhYABBY4_Dt | Gh_D01G2326 | - | scaffold3715_D01 |
| GhYABBY5_Dt | Gh_D01G1535 | 0 | D01 |
| GhYABBY6_Dt | Gh_D02G1305 | 0 | D02 |
| GhYABBY8_Dt | Gh_D11G0842 | 0 | D11 |
| GhYABBY9_Dt | Gh_D12G2170 | 0 | D12 |
| GhYABBY10_Dt | Gh_D05G0144 | 4 | D05 |
| GhYABBY11_Dt | Gh_D05G3293 | 0 | D05 |
| GhYABBY12_Dt | Gh_D06G0514 | 4 | D06 |

0, 1, 2, 3, 4 stand for singleton, dispersed, proximal, tandem, segmental.
