# Supplementary material for: Genome-Wide Study of YABBY Genes in Upland Cotton and Their Expression Patterns under Different Stresses
Source: Front Genet. 2018 Feb 7;9:33. doi: 10.3389/fgene.2018.00033 (PMC5808293; doi:10.3389/fgene.2018.00033)
Supplement: Supplementary file 6 [file Table6.DOCX]

**Supplementary Table 6.** Detection of positive selection under the site model. a. Average values of dN/dS (ω) under the M0 model. b. ω values estimated under the M8 model, p1 is the inferred proportion of positively selected sites; p and q are parameters of the β distribution. d. The digits before the letter are the amino acid positions, and the decimals after the letter are posterior probabilities.

| **Gr**oup | d_N_/d_S_ (ω) under M0 ^a^ | 2△LnL M3 vs M0 | 2△LnL  M2a vs M1a | 2△LnL  M8 vs M7 | M8 estimate ^b^ | Positive selective sites ^c^ |
| --- | --- | --- | --- | --- | --- | --- |
| FIL-like | 0.11719 | 143.89** | 0 | 18.06** | p1 = 0.04084,ω = 1.12618 | 16 F 0.560,18 A 0.592 |
|  |  |  |  |  | β (p=0.43144,q=3.01636) |  |
| YAB5-like | 0.17744 | 31.97** | 0 | 8.95** | p1= 0.03121, ω =1.15796 | 81 N 0.700 |
|  |  |  |  |  | β (p=0.80092,q=3.79953) |  |
| YAB2-1-like | 0.16128 | 27.62** | 0 | 0.574 | p1=0.2198, ω =1.0000 | 51 S 0.533,71 Y 0.573,79 Q 0.629,82 S 0.734,85 L 0.645,86 R 0.565,89 P 0.590 |
|  |  |  |  |  | β (p=0.04480,q=0.49204) |  |
| YAB2-2-like | 0.2413 | 252.12** | 0 | 0.00012 | p1 = 0.0001, ω =1.0000 |  |
|  |  |  |  |  | β (p=0.3427,q=0.7196) | NA |
| YAB2-3-like | 0.2952 | 48.6** | 0 | 2.14 | p1 = 0.0107, ω =6.64756 | 1 M 0.736,133 G 0.867 |
|  |  |  |  |  | β (p=05787,q=1.19) |  |
| INO-like | 0.30511 | 34.4** | 0 | 1.06 | p1 = 0.0114, ω =2.99615 | 51 A 0.785 |
|  |  |  |  |  | β(p=1.3180,q=2.6746) |  |
| CRC-like | 0.1064 | 132.56** | 0 | 3.86 | p1= 0.03871, ω =1.55 | 3 L 0.605,73 T 0.574,85 S 0.531,149 C 0.882 |
|  |  |  |  |  | β (p=0.4178,q=3.2349) |  |
