# Supplementary material for: Genome-Wide Study of YABBY Genes in Upland Cotton and Their Expression Patterns under Different Stresses
Source: Front Genet. 2018 Feb 7;9:33. doi: 10.3389/fgene.2018.00033 (PMC5808293; doi:10.3389/fgene.2018.00033)
Supplement: Supplementary file 9 [file Table9.DOCX]

| Motif | Expression Pattern | Width | Nsite | E-value |
| --- | --- | --- | --- | --- |
| 1 | PPEKR[QH]R[VA]PSAYN[RQ]FIKEEIQRIKA[NG]NPDISH REAFS[TA]AAKNWA[HR]FP[HP]IHFGL[MK][LP][ED][GQT] | 57 | 29 | 8.2e-1325 |
| 2 | [SM][DCS][HL]NS[AP][PS][ED]QLCY[VI][HP]CNFC[ND] [TI][VI]LAV[SN]VPC[SNT]SLF[DKE] | 33 | 29 | 5.1e-552 |
| 3 | [TI]VTVRCGHCTNL[LW]SVNM[AR][AG][ASL][LF] | 21 | 29 | 3.7e-382 |
| 4 | QS[LH]S[GW][QH][DS][IF][QF][AT]P[NQ][YN][AT]L [ES][ED]YRSD | 21 | 23 | 3.80E-100 |
| 5 | [NE][ETN][IAT][TP][EKQ][EP][RP]VVNR | 11 | 18 | 1.80E-54 |
| 6 | [RH]QQEGE[ED][MAV][VL][MV]KDGFF | 15 | 8 | 1.60E-47 |
| 7 | [EG][SG][SG]SS[SK][CSK][CK]NK[FL][PS][KM]R[PA] | 15 | 17 | 1.30E-39 |
| 8 | [NK]Q[PA]KL[ND][DQ][DV]S[EAT][ED][HQ][GFI]XQ | 15 | 11 | 9.10E-26 |
| 9 | [ANT]NVGV[TS]PY | 8 | 8 | 7.20E-17 |
| 10 | [FS][CH][EL][PQR][DH][EN][FG][EF][AT][PS][SW] [HN][NV][EL][AL][ET]E[IV][GS][FN][KP] [IS][IP][CN][IP][FL]L[GN][DQ][AG][NLV][GT][NS] [DE][IV][FT][LT][AP][ST][LR][GY] | 41 | 4 | 1.70E-16 |
| 11 | [NTQ][MNI][MVH][MVY]I[NY][HQI][PY]N[PG][NK][ND] [STV][LV][IM][PRM][GP][AIF][AHG][EG][GL] | 21 | 5 | 1.80E-10 |
| 12 | L[MV]SSSDN[GE][ED]ED[IV][VS]RV[NY][PQ][TV]VNK | 21 | 3 | 7.20E-08 |
| 13 | EA[PT]RMPPI | 8 | 4 | 1.50E-04 |

**Supplementary Table 9. The regular expression for motifs identified by MEME**
