# Supplementary material for: Genome-Wide Study of YABBY Genes in Upland Cotton and Their Expression Patterns under Different Stresses
Source: Front Genet. 2018 Feb 7;9:33. doi: 10.3389/fgene.2018.00033 (PMC5808293; doi:10.3389/fgene.2018.00033)
Supplement: Supplementary file 10 [file Table10.DOCX]

| Primer name | Sequence (5’-3’) | Application |
| --- | --- | --- |
| GhYABBY1_At-F | GCGGCTGCATTTCAGTCAC | qPCR(Candidate genes) |
| GhYABBY1_At-R | GCTCGCATTGAGAACTTGGTGT |  |
| GhYABBY2_At-F | CAGTCAAGATGCTCAGAAACAGCA |  |
| GhYABBY2_At-R | GGGGGCGAATAGGTGGCATT |  |
| GhYABBY3_At-F | GCTGCTTTTTCACCGGACCA |  |
| GhYABBY3_At-R | GCGAGGAACACTCACAGCGA |  |
| GhYABBY4_At-F | GTGGGCAGTGCACCAATCT |  |
| GhYABBY4_At-R | TTGCACCTGGAGGAAGACCC |  |
| GhYABBY5_At-F | ACAGTCAACAAGCCGCCAG |  |
| GhYABBY5_At-R | TCTTTGTGCGGAATGTTGGG |  |
| GhYABBY6_At-F | TTCTCCAGCAGCATCATCCGTT |  |
| GhYABBY6_At-R | CCCTTCGCCAAATCCCATAAT |  |
| GhYABBY7_At-F | TCATCCCCTAAAGCGCCATTTG |  |
| GhYABBY7_At-R | ACGGATGATGCTGCTGGAGAAT |  |
| GhYABBY8_At-F | GTGCCGTCTGCCTACAATCG |  |
| GhYABBY8_At-R | TTGGCAGCAGCACTGAAGGCG |  |
| GhYABBY9_At-F | CCCCTCAGCGTACAACCGAT |  |
| GhYABBY9_At-R | CTTGCTGGCGCACGTTAGTCC |  |
| GhYABBY10_At-F | TCGTTCTTGCGGTGAGTGTCC |  |
| GhYABBY10_At-R | GCTGCCATGTTCACGGACC |  |
| GhYABBY11_At-F | TGACAAGAGGCATAGGGCA |  |
| GhYABBY11_At-R | TGAATGTGAGGGAAGCGTG |  |
| GhYABBY12_At-F | GACCTCCTCTGCCTAACA |  |
| GhYABBY12_At-R | TACTCGCTGCCTCTTCTCGG |  |
| Ghhiston3-F | TCAAGACTGATTTGCGTTTCCA | qPCR (Internal control) |
| Ghhiston3-R | GCGCAAAGGTTGGTGTCTTC |  |

**Supplementary Table 10. Sequences of primers used for qPCR**
