# Supplementary material for: Genome-Wide Study of YABBY Genes in Upland Cotton and Their Expression Patterns under Different Stresses
Source: Front Genet. 2018 Feb 7;9:33. doi: 10.3389/fgene.2018.00033 (PMC5808293; doi:10.3389/fgene.2018.00033)
Supplement: Supplementary file 11 [file Image1.PDF]

Supplementary Image 1. Comparative analysis of *GhYABBT7_At* and *GhYABBT7_Dt* indicating that gaps disrupting the GhYABBY7_At.

```
D09_35584089-35     1 NNNNNNNNNNNNNNNNNNNNNNNNNNNNNNNNNNNNNNNNNNNNNNNNNNNNNNNNNNNN
GhYABBY7_At         1 ATGTCACTGGATTTGGCATCTGAACGTGTTTGTTATGTTCACTGCAACTTCTGCAACACC

D09_35584089-35    61 NNNNNNNNNNNNNNNNNNNNNNNNNNNNNNNNNNNNNNNNNNNNNNNNNNNNNNNNNNNN
GhYABBY7_At        61 ATTTTAGCGGTACACCACTCTAATTCTGATTTTCTTTTTCTCTGTTTATATAGCTTTTCT

D09_35584089-35   121 NNNNNNNNNNNNNNNNNNNNNNNNNNNNNNNNNNNNNNNNNNNNNNNNNNNNNNNNNNNN
GhYABBY7_At       121 TTTTTTGTTTTTGGGAAAAGAGAGTTGAATTGCCCATGACTGGCTTGTTTTCAATGCCTA

D09_35584089-35   181 NNNNNNNNNNNNNNNNNNNNNNNNNNNNNNNNNNNNNNNNNNNNNNNNNNNNNNNNNNNN
GhYABBY7_At       181 CCCTTTTGGTTTTGCTGAATTGGGTTCTTTTCTTGAAGAACAGTGTGTGGGAATTCACCT

D09_35584089-35   241 NNNNNNNNNNNNNNNNNNNNNNNNNNNNNNNNNNNNNNNNNNNNNNNNNNNNNNNNNNNN
GhYABBY7_At       241 GAAAATATTTAACACTTAAAATATGCCCTTTTACCCCTCCAATTTTCTTCTTCTTAATTT

D09_35584089-35   301 NNNNNNNNNNNNNNNNNNNNNNNNNNNNNNNNNNNNNNNNNNNNNNNNNNNNNNNNNNNN
GhYABBY7_At       301 TATGGCCAAAAACAAAAAAACACGCAAAAATAATATTCACTTTACCTATCATAATTCACG

D09_35584089-35   361 NNNNNNNNNNNNNNNNNNNNNNNNNNNNNNNNNNNNNNNNNNNNNNNNNNNNNNNNNNNN
GhYABBY7_At       361 GATCATGTGATTTCTTGATTTGACTTGGAAAAAGTAGCTTAAAATTCTTCTTCTTATGTC

D09_35584089-35   421 NNNNNNNNNNNNNNNNNNNNNNNNNNNNNNNNNNNNNNNNNNNNNNNNNNNNNNNNNNNN
GhYABBY7_At       421 ATATTTTATGAGAAATTTTAGCCTGGTTGGGGATTTTATGATTTGAAACTCTTGAAAAGA

D09_35584089-35   481 NNNNNNNNNNNNNNNNNNNNNNNNNNNNNNNNNNNNNNNNNNNNNNNNNNNNNNNNNNNN
GhYABBY7_At       481 TTGATCCCTCATAAACAAGCAAATGAAGTGAGTTTCAACAGTTCAAAAGGGAAAACAAAA

D09_35584089-35   541 NNNNNNNNNNNNNNNNNNNNNNNNNNNNNNNNNNNNNNNNNNNNNNNNNNNNNNNNNNNN
GhYABBY7_At       541 TATCCTCCAAAGGGACTTGTTCTGGTGGTAAAAGCTTTTTCAGGCTATGACTAGGGCTAC

D09_35584089-35   601 NNNNNNNNNNNNNNNNNNNNNNNNNNNNNNNNNNNNNNNNNNNNNNNNNNNNNNNNNNNN
GhYABBY7_At       601 AGGTAAGCACTATTTAGGGTTCTTCCAGGTCAAGGTACAAGTCAACAATGGAGGAGATTC

D09_35584089-35   661 NNNNNNNNNNNNNNNNNNNNNNNNNNNNNNNNNNNNNNNNNNNNNNNNNNNNNNNNNNNN
GhYABBY7_At       661 TTTCCTTTTCCTACCTTCCAAAGTCAATATTATTCATGGTACTTCATGTCTGATTTACCG

D09_35584089-35   721 NNNNNNNNNNNNNNNNNNNNNNNNNNNNNNNNNNNNNNNNNNNNNNNNNNNNNNNNNNNN
GhYABBY7_At       721 CTCCTCTCCTCTCCAAATTCAAGCCTACAAGTTGCTTTAGGTTTTTTCAATTACAAATCC

D09_35584089-35   781 NNNNNNNNNNNNNNNNNNNNNNNNNNNNNNNNNNNNNNNNNNNNNNNNNNNNNNNNNNNN
GhYABBY7_At       781 TCTTAGATCTTCTTCATTTTATCAGCTAAACATCTACTGCATCAACAATCTTTCTTCTTT

D09_35584089-35   841 NNNNNNNNNNNNNNNNNNNNNNNNNNNNNNNNNNNNNNNNNNNNNNNNNNNNNNNNNNNN
GhYABBY7_At       841 TAAGATCTTTACTCTTATGGGTGCTTTTTGTGATGGACATGTATTCGATATAAATATCAA

D09_35584089-35   901 NNNNNNNNNNNNNNNNNNNNNNNNNNNNNNNNNNNNNNNNNNNNNNNNNNNNNNNNNNNN
GhYABBY7_At       901 AAAGAAAGAGGTGGAGAGAGAGGTGGTCATGGATAATAAGCAGCAAGCAATAAAACCCAA

D09_35584089-35   961 NNNNNNNNNNNNNNNNNNNNNNNNNNNNNNNNNNNNNNNNNNNNNNNNNNNNNNNNNNNN
GhYABBY7_At       961 GTGCTGCTTTTCTTTTTCAACCATCTTTTCCTCATAACATGCTCCGAAAACAAACAAAAC

D09_35584089-35  1021 NNNNNNNNNNNNNNNNNNNNNNNNNNNNNNNNNNNNNNNNNNNNNNNNNNNNNNNNNNNN
GhYABBY7_At      1021 CACATTTACAGGATCCCAAACACTGCACATAGATCAGTAAATCAATCACCATTCATGATA
```

```
D09_35584089-35  1081  NNNNNNNNNNNNNNNNNNNNNNNNNNNNNNNNNNNNNNNNNNNNNNNNNNNNNNNNNNNN
GhYABBY7_At      1081  AATGTTTTTTTTAATCTTTTTCTTTATTCAATGGCCATATTTGATAGCTCATGACTTTTA

D09_35584089-35  1141  NNNNNNNNNNNNNNNNNNNNNNNNNNNNNNNNNNNNNNNNNNNNNNNNNNNNNNNNNNNN
GhYABBY7_At      1141  TGGTTTCCGTTCCAGTCACGTCGTTTTCATCTCTCTCTTTCACTCTGGGTTTTCTTTTTT

D09_35584089-35  1201  NNNNNNNNNNNNNNNNNNNNNNNNNNNNNNNNNNNNNNNNNNNNNNNNNNNNNNNNNNNN
GhYABBY7_At      1201  TCCTTTTTCTATCTTTTATCATCTAGCTTGCGGTGTCAGCCAAAAAAGTGAGGCGCACCG

D09_35584089-35  1261  NNNNNNNNNNNNNNNNNNNNNNNNNNNNNNNNNNNNNNNNNNNNNNNNNNNNNNNNNNNN
GhYABBY7_At      1261  AAAGGAAAATACATAATAATATTGGGGGAGAAATGAGAAGTCCTTTGTACTTGTAGTCAT

D09_35584089-35  1321  NNNNNNNNNNNNNNNNNNNNNNNNNNNNNNNNNNNNNNNNNNNNNNNNNNNNNNNNNNNN
GhYABBY7_At      1321  TGCCCATGTGAGCTAATCAAATCACTCACCTCTTTCAACTTTGATGTCTTATTAGTATAA

D09_35584089-35  1381  NNNNNNNNNNNNNNNNNNNNNNNNNNNNNNNNNNNNNNNNNNNNNNNNNNNNNNNNNNNN
GhYABBY7_At      1381  ACTTGGCACCTGCTATCATTTCTATCTCCTCCCCCATATAAATAGAACCCTCAATGAAAA

D09_35584089-35  1441  NNNNNNNNNNNNNNNNNNNNNNNNNNNNNNNNNNNNNNNNNNNNNNNNNNNNNNNNNNNN
GhYABBY7_At      1441  CTGAAAGCCATACGTAGTTTTTATGACAAAGGGTTACTTCATGTTCATGACTCCCCCCCC

D09_35584089-35  1501  NNNNNNNNNNNNNNNNNNNNNNNNNNNNNNNNNNNNNNNNNNNNNNNNNNNNNNNNNNNN
GhYABBY7_At      1501  CCCTTTCTCTCATTTGCAAGTGTTTGGATACTCAGAAAATGGCAGATCCGGGGACCATAG

D09_35584089-35  1561  NNNNNNNNNNNNNNNNNNNNNNNNNNNNNNNNNNNNNNNNNNNNNNNNNNNNNNNNNNNN
GhYABBY7_At      1561  AAGGAAAATGTCATGGTCTCTGGGGATATTAGGCTTGGAACTTTGATTTCTTCGCTTAGG

D09_35584089-35  1621  NNNNNNNNNNNNNNNNNNNNNNNNNNNNNNNNNNNNNNNNNNNNNNNNNNNNNNNNNNNN
GhYABBY7_At      1621  GTTTGATAATATTGTAATATATGAAACAAACATAACACACCCATATATATATACAGTTTG

D09_35584089-35  1681  NNNNNNNNNNNNNNNNNNNNNNNNNNNNNNNNNNNNNNNNNNNNNNNNNNNNNNNNNNNN
GhYABBY7_At      1681  TTGCTTTTAATCAGTTCTCACTACTTTATTAAGCTTCCAAAATGGTAACATTTTGCAGGT

D09_35584089-35  1741  NNNNNNNNNNNNNNNNNNNNNNNNNNNNNNNNNNNNNNNNNNNNNNNNNNNNNNNNNNNN
GhYABBY7_At      1741  TAGTGTTCCATACAACAGCTTGTTCAAATTTGTGACTGTTAGATGCGGGCATTGTGCCAA

D09_35584089-35  1801  NNNNNNNNNNNNNNNNNNNNNNNNNNNNNNNNNNNNNNNNNNNNNNNNNNNNNNNNNNNN
GhYABBY7_At      1801  TCTGCTGTCTGTAAATATGGGAACTTCACAGCAAACAGTTCCCATTCAAGATGCTCAGGT

D09_35584089-35  1861  NNNNNNNNNNNNNNNNNNNNNNNNNNNNNNNNNNNNNNNNNNNNNNNNNNNNNNNNNNNN
GhYABBY7_At      1861  ATAAATATAATCAAGGTTCTATTTCCCCCCTCTTATCACTTCCTGAATATTAAACTCAAT

D09_35584089-35  1921  NNNNNNNNNNNNNNNNNNNNNNNNNNNNNNNNNNNNNNNNNNNNNNNNNNNNNNNNNNNN
GhYABBY7_At      1921  ACTAAAAGATTATGGAAAAGAAGGGTAAGCATTTAAGACAAGGAATGCATATTTTTTCAT

D09_35584089-35  1981  NNNNNNNNNNNNNNNNNNNNNNNNNNNNNNNNNNNNNNNNNNNNNNNNNNNNNNNNNNNN
GhYABBY7_At      1981  TAACCAAAGATTCCAAAGCATACTATTATTTTATGGGTTCCTAACCCTTTTAGCTGATAT

D09_35584089-35  2041  NNNNNNNNNNNNNNNNNNNNNNNNNNNNNNNNNNNNNNNNNNNNNNNNNNNNNNNNNNNN
GhYABBY7_At      2041  TAATAATATTATACTGTGCTGTGTTCTCAAAATACTATATGAAACAATAAATCTGTTGTT

D09_35584089-35  2101  NNNNNNNNNNNNNNNNNNNNNNNNNNNNNNNNNNNNNNNNNNNNNNNNNNNNNNNNNNNN
GhYABBY7_At      2101  TCAGTTGATTTGACCTCTTTCCTCTTATACTCTGATATTATTATACATACTAACCCTTTT
```

```
D09_35584089-35 2161 NNNNNNNNNNNNNNNNNNNNNNNNNNNNNNNNNNNNNNNNNNNNNNNNNNNNNNNNNNNN
GhYABBY7_At     2161 AGTTTCTTTTGTTGGGTGTTTTTCGTTACAATTAATATATGTACCTTGGTCTTTGGATGT

D09_35584089-35 2221 NNNNNNNNNNNNNNNNNNNNNNNNNNNNNNNNNNNNNNNNNNNNNNNNNNNNNNNNNNNN
GhYABBY7_At     2221 GTTTTTTAAACCAAGGCCAAAGTGGAGCTTCAATGCTCTTCTCAATGCTATCAAAAGCTT

D09_35584089-35 2281 NNNNNNNNNNNNNNNNNNNNNNNNNNNNNNNNNNNNNNNNNNNNNNNNNNNNNNNNNNNN
GhYABBY7_At     2281 TGTGGGCCAAATCAGAAAGGGACTGTTTGGAGGATTCACTTGTTTACTTTGTTTTGAGTG

D09_35584089-35 2341 NNNNNNNNNNNNNNNNNNNNNNNNNNNNNNNNNNNNNNNNNNNNNNNNNNNNNNNNNNNN
GhYABBY7_At     2341 GAGTTGCAGAAACAGCAGATCATAAACATTGATGAAGATCCTTCAAATAAGGAATGTGGG

D09_35584089-35 2401 NNNNNNNNNNNNNNNNNNNNNNNNNNNNNNNNNNNNNNNNNNNNNNNNNNNNNNNNNNNN
GhYABBY7_At     2401 TCGTCTTCCAAATGCAACAAGCAGTTTTCTGGATTTGACTCTGCACAAAATGAAGCACCC

D09_35584089-35 2461 NNNNNNNNNNNNNNNNNNNNNNNNNNNNNNNNNNNNNNNNNNNNNNNNNNNNNNNNNNNN
GhYABBY7_At     2461 AGAATGCCTCCTATTCGCCGTTAGTCTCTCTCCCTCTCATATATAGTATTTATATTTGAT

D09_35584089-35 2521 NNNNNNNNNNNNNNNNNNNNNNNNNNNNNNNNNNNNNNNNNNNNNNNNNNNNNNNNNNNC
GhYABBY7_At     2521 GGACTATCAAATTCTATGCTCTCTTAGTTCACTGATAATACATCAAATTTTAACTCATAT

D09_35584089-35 2581 AAGTAATCCAGACAT-CAGCCACAGGGAAGCTTTCAGCACTGCTGCAAAAAATGTAAGTT
GhYABBY7_At     2581 ATATTGTACTTATACATATCTACGCAAATGCATTGAGCTAAGTTCCTTA---TGCAAAAG

D09_35584089-35 2640 TTTGGGATACATACAATATATACTCATTGCTTATCTAATGCAATTAACCATAACTCCATT
GhYABBY7_At     2638 CCCA--ATGCATAGTTGATACCATAAGGTTAAATGTAGAACCAAATACC-TAGCTTTGT

D09_35584089-35 2700 ATAAGAAATCTTTTCTTCTTCTTGTTGTTGTTTTAAGTTGATGGGTCGAACTTACTCTTT
GhYABBY7_At     2695 ATGACGGATATGGAAAACTTGGAACCAAAAGGATAA---AAAGGGGCTCCCTCACAACTG

D09_35584089-35 2760 TTAAGAAGAAATAAATTTG---ATAAAAAAAATATCTACTTCCTCCTAAAGTTTGAAAA
GhYABBY7_At     2752 GCAAAAGTAAAAAAATCAACACATTAAGCAACTTATCTGAGCTCTCCT----TTCCAATC

D09_35584089-35 2817 ATACCCACTTATCTCCTAAAGTTTGATAAAAATACATTTACTTATTAAACAAATTGTAAA
GhYABBY7_At     2808 ATAGCC---------CCTACAAGATGGTGACCTTACATA-ACAAGTTTAACTCCTCATTAA

D09_35584089-35 2877 TAAATAAAAACTTCCCTTTTAATAATTTCTTATTTTTTAATTTGTTCTTGTTATCTTCA
GhYABBY7_At     2859 TTAT-TAACAGN--ACTTTTTATTAATTACTTATTTTTTAATTTGTTCTTGTTATCTTCA

D09_35584089-35 2937 ATCTTTATTTTCAATAATTTTAAATTAAAAATAATAAAAACATAATTAATTATGAAATAT
GhYABBY7_At     2916 ATCTTTATTTTCAATAATTTTAAATTAAAAATAATAAAAA-ATAATTAATTATGAAATAT

D09_35584089-35 2997 TTTAATTATTATTAAATTAAATTATAAATTATTTTGTAATCATATGTTAAACATAATGTA
GhYABBY7_At     2975 TTTGATTATTATTAAATTAAATTATAATTTTTTTTGTAATCATATGTTAAACATGATATA

D09_35584089-35 3057 TTTTTCAATTAATATATACAAGTTTTTCTTGAGATTACAAATACATTCACATTGTAACTA
GhYABBY7_At     3035 TTTTTCAATTAATATATACAAGTTTTTCTTGAGGTTACAAATACATTCACATTGTAATTA

D09_35584089-35 3117 CAAAGAATAGATCGGATAGCCTTAATCATTATAAAATACGATTAGATTAATAACCAAAAT
GhYABBY7_At     3095 CAAAGAATAGATCGGATAGACTTAATCATTATAAAATACGATTAGATTAATAACCAAAAT

D09_35584089-35 3177 ATGATCCAGGCTTATATCAAAACAAATTAAATTATAAATTGTTTTATATTTTAAAATAAT
GhYABBY7_At     3155 ATGATGCAGGCTTATATCAAAACAATTTAATTTATAAATTGTTTTATATTATAATATAAT
```

```
D09_35584089-35  3237  CATATTTTAAATATAATGTATTTTCAATTAATATATTAAATAAATTTTTTTAAGTTAAAA
GhYABBY7_At      3215  CATATTTTAAATATAATGTATTTTCAATTAATATATTAAATAAATTTTTTTAAGTTAGAA

D09_35584089-35  3297  ATACATTGCGATTAATAAAAAATAGATTGGAATAACTATAACGATAATTAATTATTATAA
GhYABBY7_At      3275  ATACATTGTGATTAATAAAAAATAGATTGGAATAACTATAATGACAGTTAATCATTACAA

D09_35584089-35  3357  AGCACGATTAATTTGATAACAAAATATATTTAAACTCAGACTAAAATAAATTTATACATG
GhYABBY7_At      3335  AGCATAATTAATTTGATAACAAAATATATCTAAACTTAGACTAGAATAAATTTATACAAG

D09_35584089-35  3417  ACCCAAATATAATAAGAAATAAAT----------ATGAATTAAGAATCTATACATGATTT
GhYABBY7_At      3395  ACCCAAATATAATAAAAAATAAATTAAAATAAATATGAATTAAAAACCTATACATGATTT

D09_35584089-35  3467  ATACAAAA-TGTACTTGAAATTGAGATGTTAAAATTATTGTGGATTTAACCTTACCCATT
GhYABBY7_At      3455  ATACAAAAATAAACTTGAAATTGAGATCTT------------GATTTAACCTTACCCATT

D09_35584089-35  3526  AATGTAAGACTTCATTGCCCGTATTTTATTTTAGCTTCTCTTTTATTTTTTTTTTCT---
GhYABBY7_At      3503  AATGTAAGACTTCATTGCCCATATTTTATTTTATCTTCTCTTTTACTCTTTTTTTTTTAA

D09_35584089-35  3583  AATAATTTGTTATGCAAATACATGTTACAACCTTTTAAAATTTAATTATATATTAATTTT
GhYABBY7_At      3563  AATAATTTCTTATGCAAATACATGTTACAACCTTTTAAATTTTAATTATATATTAATTTT

D09_35584089-35  3643  GAGATTTTTT-ATATTTTAGTATTTTTTTCCATTAAAACTTTTTGAATAATGGTAACTTA
GhYABBY7_At      3623  GAGATTTTTTTATATTTTAGTATTTTTT-CCATTAAAACTTTTTGAATAATGGTAACTAA

D09_35584089-35  3702  TTGTTTTACATAGAATTCATCTTAAGGTTAACATATAACTAAATTAAATATAAAATTTAA
GhYABBY7_At      3682  TTATTTTACATAGAATTCATCTTAAGCTTAACATATAACTAAATTA--TATAAAATTAAA

D09_35584089-35  3762  ATACCTAATTTAAATTGCAGTCAAATATAATTTTTATAATAAAAT------ATATTTAAT
GhYABBY7_At      3740  ATACTTAATTTAAATTGAAGTCAAATATAATTTTTATAAAAAAATTTAAATATATTTAAT

D09_35584089-35  3816  ATTATAATACTTCAATATGTAATTAAATTTTA----TTTATTTTTTATTTTTTATACAAT
GhYABBY7_At      3800  ATTATAATACTTCAATATGTAATTAATTTTTTATTTTTTATTGTTTATTTTTTATACAAT

D09_35584089-35  3872  CAATTTATGCCTACTTCTACCATAACCTTTCTCAACATGTAATTGAACCCGTGTCCTCTT
GhYABBY7_At      3860  CAATTTATGCCTACTTCTACCACAACTCTTCTCAAGATTTAATTGAACCCGTGTCCTCTT

D09_35584089-35  3932  GATTAACTTGTAATTAAAACTATAAAAATAAATAAAAAATAGAAAATATATAGATAAATA
GhYABBY7_At      3920  AATTAACTTGTAATTAAAACTATAAAAATAA---AAAAATAGAAAATATATAGATAAATA

D09_35584089-35  3992  GTGACAATATTTTTATTATTTTATCTTTTCATTGAAAAATTGCTTAGCTAATAGATCTAA
GhYABBY7_At      3977  ATGACAATATTTTTATTATTTTATCTTTTCATTTAAAACTTGCTTAGCAAATAGATCTAA

D09_35584089-35  4052  CGGTTTATAGAATTTTCTTAAAATAGGAGGCATAAGGGCATTCTAAAAATCATAGGGATA
GhYABBY7_At      4037  CAGTTTATATGATTTTCTTCAAATATGAGGCATAAGGGCATTCTAAAAATCATAGGGATA

D09_35584089-35  4112  TGTGTATTTTACACTAAATTAGGATTAAGTTATTTTTGAA---------------GATTT
GhYABBY7_At      4097  TGTGTAATTTACAGTAAATTAGGATTAAGTTATTTTTTAATACCCAAAACATTATGATTT

D09_35584089-35  4157  TTAATTTTAATTTTATCATTTCAACTAAAGTTATATTCGATTATTATAATTTTTTTAAAA
GhYABBY7_At      4157  TTAGTTTTAATTTTACCATTTTAACTAAACTCATATTTGATTATTATAATTTTTTTAAAA

D09_35584089-35  4217  AAATTATTATATACATTTTTTATCCATGTCGTGATTGTGTTATAATCTATTATATAGATA
GhYABBY7_At      4217  --ATTATTATATATATTTTTTATTCAAGTCGTGAGTGTGTCATAATCTATGATATAGGTA
```

```
D09_35584089-35  4277  TAAATAAAAA-ATATATTAAAAAATACTTAAACATTATCCATTTATCCAATGAATAATAC
GhYABBY7_At      4275  TAAATAAAAACATAT-TCAAAAAATACTTAAACATTATCCATTTATCCAATGAATAATAC

D09_35584089-35  4336  ACACCTTTCGTAATGAAATTACGTCCCTAATGCCAGGCTATTAATTTGG-----------
GhYABBY7_At      4334  ACACCTTTCGTAATGAAATTACGTCCCTAATGCCAGGCTATTAATTTGGTTATATCCACA

D09_35584089-35  4385  ------GTTGAGAGCTAATGATTTGTTCTCCACATGTTGAAGGAAAGAAGAAATGTAGGA
GhYABBY7_At      4394  ATTTGTGTTGAGAACTAATGATTTGTTCTCCACATGTTAAAGGAAACAAGAAATGTAGGA

D09_35584089-35  4439  CTTTTGCCATGCCTTGAAAATATCTTCCACATCCTGTCTTTATATTGACAAGTGAAGAAA
GhYABBY7_At      4454  CTTTTGCCATGCCTTGACAATATCTTCCACATCTTATCTTTATATTGACAAGTGAAGAAA

D09_35584089-35  4499  CAATTTAGCCCCGATTATTTGGGTTTGACTTCTTATATGAATATGTGTTTATATTGGAAG
GhYABBY7_At      4514  CAATTTAGTCCCTATTATTTGGGTTTCACTTCTTATATGAATATGTATTTATATTGGAAG

D09_35584089-35  4559  ATTATAAGTGTATTTTAGAAGACTTGCACTAATCCTTCCCAAAGACATGATAGAATGCAA
GhYABBY7_At      4574  ATTATAAGTGC--TTTAGAAGACTTGCACTAAT-------------ATGATAGTATGCAA

D09_35584089-35  4619  ATAACAACATAAATGCTTTTGCAGTGGGCACATTTTCCTCACATTCACTTTGGGCTAAAA
GhYABBY7_At      4619  ATAACAACATAAATGCTTTTGCAGTGGGCACATTTTCCTCACATTCACTTTGGGCTAAAA

D09_35584089-35  4679  CTGGAGGGAAACAAGCAAGCAAAACTGGACCAGTCATTTGCAGACCAAGGTGCTCAAAAG
GhYABBY7_At      4679  CTGGAGGGAAACAAGCAAGCAAAACTGGACCAGTCATTTGCAGACCAGGGTGCTCAAAAG

D09_35584089-35  4739  TCTAACAACTACTACTGA
GhYABBY7_At      4739  TCTAACCCCTACTACTGA
```
