# Supplementary material for: Genome-Wide Study of YABBY Genes in Upland Cotton and Their Expression Patterns under Different Stresses
Source: Front Genet. 2018 Feb 7;9:33. doi: 10.3389/fgene.2018.00033 (PMC5808293; doi:10.3389/fgene.2018.00033)

Supplementary Image 2. Phylogenetic tree of YABBY genes indicating that YABBY genes can be clustered into five groups. MEGA software version 7.0 was used for constructing the tree using the minimum-evolution method. The bootstrap values are shown near the nodes, and only values >50 are displayed.

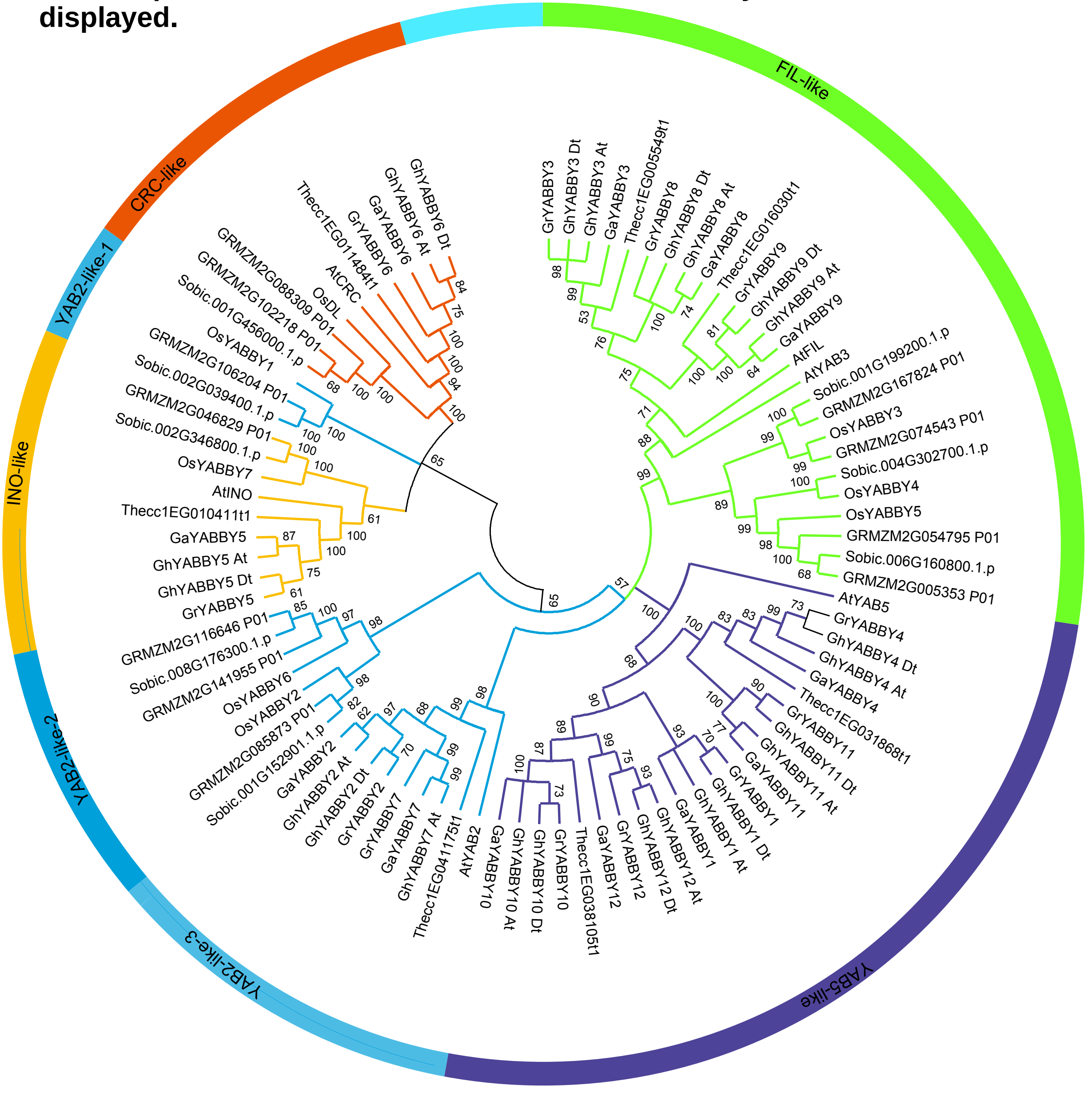

Supplement: Supplementary file 12 [file Image2.PDF]
