# Supplementary material for: Genome-Wide Study of YABBY Genes in Upland Cotton and Their Expression Patterns under Different Stresses
Source: Front Genet. 2018 Feb 7;9:33. doi: 10.3389/fgene.2018.00033 (PMC5808293; doi:10.3389/fgene.2018.00033)
Supplement: Supplementary file 13 [file Image3.PDF]

Supplementary Image 3. Comparative analysis of GhYABBT6_At and GhYABBT6_Dt indicating that a premature stop leads to sequence divergence between the orthologous pair.

```
GhYABBY6_At    1 ATGAACCTTGAAGACAAAGTTGGGATGGACTTGGTTCCACAATCCGAGCATCTTTGCTAT
GhYABBY6_Dt    1 ATGAACCTTGAAGACAAAGTTGGGATGGACTTGGTTCCACAATCCGAGCATCTTTGCTAT

GhYABBY6_At   61 GTCCGCTGCAACTTCTGCAACACTGTTCTTGCGGTATAATTTCACTCCCAATATGGAGCT
GhYABBY6_Dt   61 GTCCGCTGCAACTTCTGCAACACTGTCCTTGCGGTATAATTTCACTCCCAATATGGAGCT

GhYABBY6_At  121 CAATGGTTATCATTGTTATTATTTATTGTAGCCGCAAATACAACGAACTGCTATTGAAAT
GhYABBY6_Dt  121 CCATGGTTATCATTATTGTTTTTTATTGTAGCTGCAAATACGACGAACTGTTATTGAAAT

GhYABBY6_At  181 CAATTGCGGCAGTTAATATCAGAGCCAGTCGCTGCGAAGCAACACGCTATTAAAATCCTT
GhYABBY6_Dt  181 CAATTGCGGCAGTTAATATCAAAGCCAGTCGCTGCGAAGCAACACGCTATTAAAATCCTT

GhYABBY6_At  241 ACTGGCTAGTACATAGATTATTAGGGTCTCGGGAGCTAACCTCATCTATAATGAAATTAA
GhYABBY6_Dt  241 ACTGGCTAGTACATAGATTATTAGGGTCTCGGTAGCTAACCTCATCTATAATGAAATTAA

GhYABBY6_At  301 GCTGCAAGTTTTGTGTTTGATGTTCTAATAAGATGCTGGTTTGGGGTTTGTATATATGTT
GhYABBY6_Dt  301 GCTGCAAGTTTTGTGTTTGATGTTCTCATAAGATGCTGGTTTGGGGTTTGTATATATGTT

GhYABBY6_At  361 TATTATGTCTCCGTAATTGTGCCCATTTTCTTGTATTTTCTTGATCTGAATTGGTTTTTG
GhYABBY6_Dt  361 TATTATGTCTCCGTAATTGTGCCCATTTTCTTGTATTTTCTTGATCTGAATTGGTTTTTG

GhYABBY6_At  421 ATAGGTTGGGATCCCATGCAAAAGATTGCTTGAAACAGTGACAGTGAAATGTGGTCATTG
GhYABBY6_Dt  421 ATAGGTTGGGATCCCATGCAAAAGATTGCTTGAAACAGTGACAGTGAAATGTGGTCATTG

GhYABBY6_At  481 CAGTAACCTTTCTTTTCTCAGCACCAGACCTCCACTGCAAGGTCAATGCCTCGATCCCCA
GhYABBY6_Dt  481 CAGTAACCTTTCTTTTCTCAGCACCAGACCTCCACTGCAAGGTCAATGCCTCGATCCCCA

GhYABBY6_At  541 AACCAGCCTCACTCTCCAGGTACGTTTTCCATTTTTAAACCAAGAGAAATAAAAGCATCA
GhYABBY6_Dt  541 AACCAGCCTCACTCTCCAGGTACGTTTTCCATTTTTAAACCAAGAGAAATAAAAGCATCA

GhYABBY6_At  601 AAGGTGGAAACCAAGTGCATGCCACCATTAGTTCTAGCATACAATTACAGTTAGGAAGCA
GhYABBY6_Dt  601 GAGGTGGAAACCAAGTGCATGCCACTATTAGTTCTAGCATACAATTACAGTTAGGAAGCA

GhYABBY6_At  661 AATCTCCATTTCTTTTCTATATATTCTTGTATTACCTTTCACAATTATGCATGCACATTT
GhYABBY6_Dt  661 AATCTCCATTTCTTTTCTATATATTCTTGTATTACCTTTCACAATTATGCATGCACATTT

GhYABBY6_At  721 ATATTCCACACCTCAAAGTAAAATAGAACAGACAAGAGTTGATTTGATTTGATTAGTAAT
GhYABBY6_Dt  721 ATATTCCACACCTCAAAGTAAAATAGAACAGACAAGAGTTGATTTGATTTGATTAGTAAT

GhYABBY6_At  781 GGAATGAAGTTTGTTCATGTCAAGGAGATGCAGAGTTTCTGCGGTGATTTCAGGAAAGGT
GhYABBY6_Dt  781 GGAATGAAGTTTGTTCATGTCAAGGAGATGCAGAGTTTCTGCGGTGATTTCAGGAAAGGT

GhYABBY6_At  841 ACTCAGTTTCCATCGCCATCTTCATCAACATCGAGCGAGCCATCATCCCCTAAAGCGCCA
GhYABBY6_Dt  841 ACTCAGTTTCCATCGCCATCTTCATCAACATCGAGCGAGCCATCATCCCCTAAAGCGCCA

GhYABBY6_At  901 TTTGTTGTAAAACGTATATATATAT------GGAAACCCTTAATAGTATCATACTCCTTT
GhYABBY6_Dt  901 TTTGTTGTAAAACGTATATATATATATATATGGAAACCCTTAATAGTATCATACTCCTTT

GhYABBY6_At  955 GATTTTCTTGCAACCCTTCTTTCTCCAGTATGTTTGGATTAATTGGAAACTGCTACTTTC
GhYABBY6_Dt  961 GATTTTCTTGCAACCCTTCTTTCTCCAATATGTTTGGATTAATTGGAAACTGCTACTTTC
```

```
GhYABBY6_At 1015 TTTTTCTTTTT-----TTCAGCCCCCGAGAAGAAACACAGGCTTCCATCTGCTTACAATC
GhYABBY6_Dt 1021 TTTTTCTTTTTCTTTTTTCAGCTCCCGAGAAGAAACACAGGCTTCCATCTGCTTACAATC

GhYABBY6_At 1070 GGTTTATGAAGTAATGAAATGTTGTTGTTTC--CTATGATACTATACGTTTTTTTTTTCT
GhYABBY6_Dt 1081 GGTTTATGAAGTAATGAAATATTGCTGTTTCTGCTATGATACTATATGTTTTTTTCTCTC
GhYABBY6_At 1128 CTCAACATCAAATTCTTTTCCCAAATTGAAAGGGAGGAAATACAGCGCATTAAAGCAGCA
GhYABBY6_Dt 1141 TCTCACATCAA-TTCTTTTCCCAAATTGAAAGGGAGGAAATACAGCGCATTAAAGCAGCA

GhYABBY6_At 1188 AATCCTGAGATACCCCATCGAGAAGCTTTCAGCGCAGCTGCTAAAAATGTAAGAGACATA
GhYABBY6_Dt 1200 AATCCTGAGATACCCCATCGTGAAGCTTTCAGTGCAGCTGCTAAAAATGTAAGAGACATA

GhYABBY6_At 1248 TATACTATTTCATCTATCTACTTGCCGTCGAGGTTTTTATTTTTCTTTTCTCTGAATAA
GhYABBY6_Dt 1260 TATACTATTTCATCTATCTACTTGCCATCGAGGTTTTTATTTTTCTTTTCTCTGAATAA

GhYABBY6_At 1308 AAACTTAAGAATACTCACCCTGTTTGTAATAAATTAGAGCTTAATTATCTTCAATTTTTA
GhYABBY6_Dt 1320 AAACTTAGGAATACCCACCCTGTTTATAATAAATTATAGCTTAATTATCTTCAATTTTTA

GhYABBY6_At 1368 TGACAATTTGGATTCTTATTCTTATTCTGACTTGCTCGGAAATGGTAACAGTGGGCTCGG
GhYABBY6_Dt 1380 TGACAATTTGGTTTCTTATTCTTATGCTGACTTGCTGGGAAATGGGAACAGTGGGCTCGG

GhYABBY6_At 1428 TACATCCCAAATTCTCCAGCAGCATCATCCGTTTGTGGAAGTAGCAGCAATGTAAGTAAC
GhYABBY6_Dt 1440 TACATCCCAAATTCTCCAGCAGCATCATCCGTTTGTGGAAGTAGCAGCAATGTAAGTAAG

                                         I  Y  N  G  K  N  V  L  I  M  G  F  G  E
GhYABBY6_At 1488 CAAATACACTATATATATATATATATAATGGTAAAAATGTTCTTATTATGGGATTTGGCGAA
GhYABBY6_Dt 1500 CAAATACACTATATATATATA----TAATGGTAAAAATGTTCTGATTATGGGATTTGGAAAA
                                             I  M  V  K  M  F  *
                  G  L  L  *
GhYABBY6_At 1548 GGGCTTCTA-TGA
GhYABBY6_Dt 1556 GGGCTGGGGCTGA
```
